# A Systematic Review and Meta-Analysis of a Measure of Staff/Child Interaction Quality (the Classroom Assessment Scoring System) in Early Childhood Education and Care Settings and Child Outcomes

**DOI:** 10.1371/journal.pone.0167660

**Published:** 2016-12-30

**Authors:** Michal Perlman, Olesya Falenchuk, Brooke Fletcher, Evelyn McMullen, Joseph Beyene, Prakesh S. Shah

**Affiliations:** 1 Applied Psychology and Human Development, University of Toronto/OISE, Toronto, Ontario, Canada; 2 Department of Gastroenterology, Alberta Children's Hospital, Calgary, Alberta, Canada; 3 Department of Clinical Epidemiology & Biostatistics, McMaster University, Hamilton, Ontario, Canada; 4 Department of Pediatrics, Mount Sinai Hospital, Toronto, Ontario, Canada; 5 Department of Pediatrics, University of Toronto, Toronto, Ontario, Canada; 6 Institute of Health Policy, Management and Evaluation, University of Toronto, Ontario, Canada; TNO, NETHERLANDS

## Abstract

The quality of staff/child interactions as measured by the Classroom Assessment Scoring System (CLASS) in Early Childhood Education and Care (ECEC) programs is thought to be important for children’s outcomes. The CLASS is made of three domains that assess Emotional Support, Classroom Organization and Instructional Support. It is a relatively new measure that is being used increasingly for research, quality monitoring/accountability and other applied purposes. Our objective was to evaluate the association between the CLASS and child outcomes. Searches of Medline, PsycINFO, ERIC, websites of large datasets and reference sections of all retrieved articles were conducted up to July 3, 2015. Studies that measured association between the CLASS and child outcomes for preschool-aged children who attended ECEC programs were included after screening by two independent reviewers. Searches and data extraction were conducted by two independent reviewers. Thirty-five studies were systematically reviewed of which 19 provided data for meta-analyses. Most studies had moderate to high risk of bias. Of the 14 meta-analyses we conducted, associations between Classroom Organization and Pencil Tapping and between Instructional Support and SSRS Social Skills were significant with pooled correlations of .06 and .09 respectively. All associations were in the expected direction. In the systematic review, significant correlations were reported mainly from one large dataset. Substantial heterogeneity in use of the CLASS, its dimensions, child outcomes and statistical measures was identified. Greater consistency in study methodology is urgently needed. Given the multitude of factors that impact child development it is encouraging that our analyses revealed some, although small, associations between the CLASS and children’s outcomes.

## Introduction

For preschool aged children, enrollment in Early Childhood Education and Care (ECEC) in many countries is now the norm [1,2]. Some studies have demonstrated that higher-quality ECEC is associated with improved language development, cognitive functioning, social competence, and emotional adjustment [3–5]. However, others have failed to report such linkages [6–8].

Structural quality indicators (e.g., staff/child ratios, staff education) generally refer to aspects of quality that are more easily quantifiable. As a result, governments tend to focus on regulation of these characteristics. Structural quality is thought to impact children indirectly by setting up the conditions in which children’s direct experiences with the environment take place [9]. For example, when staff-child ratios are better, staff may have more opportunities to have positive interactions with children. Process quality is harder to assess as it focuses on the quality of interactions that children experience directly such as the warmth and responsiveness of the staff that care for children. At least in part due to increasing calls for accountability in education systems, substantial attention is being paid to identifying the kinds of environments that support young children’s learning. Recent studies have focused attention on the quality of interactions children experience and are, therefore, thought to influence children more directly [8,10]. This focus on interactions is driven by some of the most fundamental theories in developmental psychology. These include, but are not limited to, the importance of children’s early social exchanged as outlined by attachment theory [11], Ecological Systems theory’s focus on the child’s interactions with his/her most immediate environment [12] and Vygotsky’s emphasis on learning through social exchanges by supportive “experts” [13]. In keeping with these theories, a key aspect of process quality is the warmth and responsiveness of interactions between staff and children, and the extent to which the interactions scaffold children’s learning and development. The Classroom Assessment Scale (CLASS) is a relatively new measure of the emotional tone and the quality of developmentally appropriate “instruction” (e.g., providing a language/literacy rich environment, opportunities for hands on exploration of natural phenomena, etc.) of ECEC classrooms in a systematic way. In this paper we present a systematic review of the CLASS, which is increasingly influential in the field and child outcomes.

Hamre et al. (2013) [14] present, and provide empirical support for Teaching Through Interactions. This is a conceptual framework that identified three key domains in teacher-child interactions. These domains are Emotional Support, Classroom Organization and Instructional Support. While this work largely discusses instruction within the formal education system it is based on theories that apply equally well to the preschool years. Specifically, in keeping with attachment [11], Emotional Support focuses on staffs’ ability to create warm, safe, responsive and predictable environments that help children because secure, autonomous learners. Classroom Organization captures staffs’ ability to structure the room in a way that minimizes behavioral problems and allows children to focus on their attention on learning. Finally, in keeping with Vygotskian thinking, Instructional Support focuses on the extent to which staff builds on children’s existing knowledge and scaffold leaning. The importance of these domains is supported by a body of research that links them to a host of child outcomes, broadly construed (see Cappella et al. [15] for a comprehensive review of child outcomes associated with these domains of interactions between teachers and children).

The CLASS is a key measure in this important research area as it is specifically designed to capture these three domains. Furthermore, the CLASS has also been central in the development of professional development programs for early childhood educators (e.g., MyTeachingPartner [16]) that are built around this fundamental view of good caregiving/instruction for young children. Thus, the contribution of this systematic review and meta-analysis lies within the current theoretical discussion about the importance of teacher-child interactions as a key driver of teacher effectiveness.

The CLASS is one of several classroom level measures of ECEC process quality. The most frequently used of these is the Early Childhood Environment Rating Scale–Revised (ECERS-R [17]). The ECERS-R is a global measure of quality that includes both structural aspects of the environment (e.g., the physical environment) and process quality (e.g., the kind of language that staff direct to children). There is a substantial body of research linking the ECERS-R to other measures of quality as well as to child outcomes [18–20]. The Observational Record of the Caregiving Environment (ORCE [21]) is another measure but research using the ORCE is largely based on one sample of approximately 1000 participants. As a result, it lacks the evidence base for extensive use or for a systematic review/meta-analysis. While the CLASS is a relatively new measure (certainly compared to the ECERS-R), it is supported by a growing body of evidence and grounded in a substantial theoretical/conceptual framework.

The growing importance of the CLASS is evidenced by the fact that it is now used in statewide ECEC program quality assurance systems in Arizona, Illinois, Maryland, Massachusetts, Minnesota, New Mexico, Oklahoma, Vermont, Virginia and Washington [22]. These assessments are used to make key funding and quality improvement decisions. Some quality assurance programs also make ratings public so that parents can use ratings in selecting placement for their children. Having access to empirically based measures of ECEC quality that are known to be associated with child outcomes is important for a range of stakeholders including parents and the people parents turn to for advice (e.g., their child’s pediatrician), funders, policymakers and others. Despite rapid increases in use of the CLASS in research and in high stakes accountability settings, associations between the CLASS and child outcomes have not been systematically reviewed.

We chose to cast a wide net regarding the child outcomes we included in this review. This reflects an understanding of the classroom context as having an impact on children that goes “beyond achievement tests” [15]. In keeping with this view, we set out to compile studies that used a broad range of outcomes and included information about young children’s very early academic outcomes (including language and early literacy skills); their health/wellbeing and their social-emotional development.

The objective of this review was to evaluate associations between CLASS domain and dimension scores in classrooms that served preschool aged children and children’s concurrent or subsequent social, emotional and cognitive outcomes. A secondary aim was to explore associations between specific CLASS domains and dimensions and specific child outcomes (e.g., Emotional Support to children’s social outcomes, Instructional Support for Learning to language and mathematics or Language Modeling to language outcomes).

## Method

### Selection of Studies

Searches for potential studies were conducted in three ways. First, the electronic databases, PsycINFO, Medline, and ERIC, were searched for papers that were published from the year each journal began until July 3, 2015. Search terms are provided in Tables A-D in S1 File. Second, websites for datasets that provide key data on ECEC quality and child outcomes were searched. Specifically, websites for the following databases were searched: Cost, Quality and Outcomes Study (CQO) [23]; Early Childhood Longitudinal Study (ECLS) [24]; Effective Provision of Pre-School Education (EPPSE) Project [25]; Head Start Impact Study (HS) [26]; National Center for Early Development and Learning (NCEDL) Multi-State Study of Pre-Kindergarten [27]; State-Wide Early Education Program Study (SWEEP) [27]; Family and Child Experiences Survey (FACES) [28]; and the National Institute of Child Health and Human Development (NICHD) Study of Early Child Care and Youth Development [29]. Finally, reference lists of identified studies were searched to locate additional studies. Searches were limited to English language articles only.

### Type of Participants

ECEC programs are generally divided into classrooms that serve children of different ages. We focused on research on classrooms that served preschool aged children (i.e., children approximately between the ages of 30 and 72 months) because much of the research has focused on this group [30,31] as it is the largest group of children cared for in a single type of ECEC setting [32]. The inclusion criteria used in this study and the rationale for each criterion are provided in Table 1.

**Table 1:** Inclusion Criteria for Systematic Review and Rationale.

Criteria	Rationale
***Child Care Type***	
Only studies that examined the impact of the quality of centre-based programs on children’s outcomes were included. Centre-based programs included daycare and preschool programs, nursery schools, pre-kindergarten programs, and Head Start programs. Studies that only examined home-based child care, or those in which home-based and centre-based could not be separated were excluded.	Center-based child care settings differ from home daycare in many ways such as ratios, group size, physical environment, curriculum, age range of children, and caregiver qualifications. As a result, quality is often measured differently for these two settings (e.g., ECERS versus FCCERS).
***Age Served***	
Studies that included preschool-aged children as the majority of participants were included. For the purposes of the meta-analysis, preschool-age was defined as ranging from 30 to 72 months.	Preschool-aged classrooms are different from infant/toddler classrooms due to the developmental stage and needs of the children in these two age groups. As a result, regulations and standards of care (e.g., ratios, physical environment, etc.) as well as daily activities (e.g., curriculum) differ between infant/toddler and preschool-aged classrooms.
***Child Outcomes***	
Studies that provided information about the association between CLASS on children’s cognitive, academic, social-emotional, health, or motor outcomes were included. Data could have been gathered from teachers, parents, and/or children themselves. Measures that focus on dyads (e.g., attachment) were excluded.	Cognitive, academic, social-emotional, health, and motor outcomes were selected because they are key predictors of children’s developmental trajectories. Measures that focus on staff-child or peer dyads were not included given that these outcomes often reflect an aspect of child care quality.
***Study Design***	
Cross-sectional and longitudinal designs were included. When multiple child outcome assessments were reported the earliest time-point following the measurement of quality were extracted.	To increase the homogeneity across the extracted data from eligible studies (i.e., increase the likelihood of meta-analysis), we focused on the earliest time-point in which child outcomes were measured following the measurement of quality in instances where multiple waves of outcome data were presented.
***Outcome Reporting***	
Studies must have presented statistical data quantifying the association between CLASS and a child outcome measure.	Studies only reporting qualitative results were not considered for this review as the domains of assessment could vary markedly between studies.
***Language***	
To be extracted studies had to be in English.	We did not have resources to systematically translate material written in other languages.

Abbreviations: ECERS = Early Childhood Environment Rating Scale; FCCERS Family Child Care Environment Rating Scale.

### Study Selection and Data Extraction

The selection of eligible documents was done in two steps that involved pairs of independent raters. First, the titles and abstracts of documents were screened and then full text articles were reviewed to determine eligibility. Discrepancies between raters were resolved through discussion and consensus. Relevant child and family characteristics, study characteristics and reported measures of association were extracted using standardized forms by two reviewers and discrepancies were resolved by consensus.

### CLASS Domains and Dimensions

The CLASS consists of three Domains: Emotional Support, Classroom Organization and Instructions Support. Each domain is made up of several dimensions. Specifically, Emotional Support is made up of Negative Climate, Positive Climate, Teacher Sensitivity and Regard for Student Perspective). Classroom Organization is made up of Behavior Management, Productivity and Instructional Learning Formats. Instructional Support is made up of Concept Development, Quality of Feedback, Language Modeling, along with Literacy Focus. Factor analysis of the earlier version of the CLASS yielded two domains: Emotional Climate and Instructional Climate [33]. Emotional Climate includes all of the dimensions of the Emotional Support domain and the Behavior Management dimension that later became part of the Classroom Organization domain. Instructional Climate is a composite of Concept Development and Quality of Feedback. Eligible studies used in this paper operationalized the instrument in various ways (see Table 2). When reported, findings for individual dimensions were gathered. This was done to explore the possibility that associations might be higher between specific dimensions that mapped onto specific outcomes more closely than between the broader domains and specific outcomes (e.g., that language modeling might have a stronger association to children’s language skills than Instructional Support). Including associations with dimensions also increased the comprehensiveness of our review of findings about the CLASS.

**Table 2:** Description of Studies Meeting Inclusion Criteria^a^.

Study^b^	Characteristics	Quality Measures M(SD)^c^	Outcome Measures M(SD)^d^	Covariates^e^
Aikens 2010[34]^m^^,^^B^ (2^nd^ doc. Hulsey 2010[35])	■ **Publication**: Report ■ **Design**: Longitudinal ■ **Dataset:** FACES 2006 ■ **Country**: United States ■ **Sample size:** class 410; child, range by analyses 2140–2931 ■ **% Female:** 48.6 ■ **Mean age:** 36–48 mo. ■ **Ethnicity:** C23%, B33%, H35%, A2% ■ **Mean maternal education**: NR ■ **Mean household income**: $19191	■ Instructional Support 1.9 (NR) ■ Language Modeling 2.1 (NR)	■ ECLS-Math 9.7 (3.19) ■ PPVT-4 107.9 (16.27) ■ Problem Behaviors 6.42 (0.26) ■ SSRS-SS 17.3 (0.21) ■ WJ-III-AP 390.3 (31.34) ■ WJ-III-LWI 323.5 (25.76)	■ **Statistics Extracted:** Beta, Effect Size ■ **Covariates:** child/family level—pretest score, age, gender, ethnicity, language, poverty, maternal education, maternal depressive symptoms; classroom level—full time class, peer social abilities, variation of peer abilities, peer abilities (PPVT), variation of peer abilities, DAP attitudes, teacher education; program level–SES, % ELL, program curriculum package, teacher turnover, teacher salary
Aikens 2012[36]^m^^,^^M^ (2^nd^ doc. Moiduddin 2012[37])	■ **Publication**: Report ■ **Design**: Longitudinal ■ **Dataset:** FACES 2009 ■ **Country**: United States ■ **Sample size:** class 391; child, range by analyses 903–1936 ■ **% Female:** 49.8 ■ **Mean age:** 36–48 mo. ■ **Ethnicity:** C45%, B66%, H73%, A3%, M11% ■ **Mean maternal education**: NR ■ **Mean household income**: NR	■ Classroom Organization 4.7 (NR) ■ Emotional Support 5.3 (NR) ■ Instructional Support 2.3 (NR) ■ Language Modeling 2.5 (NR) ■ Positive Climate 5.3 (NR)	■ **Age 3/Age 4** ■ BPI-PB 4.7 (.20) / 3.9 (.20) ■ ECLS-B 49.1 (20.1) / 66 (23.7) ■ ECLS-K-PB 1.8 (0.0) / 2.0 (0.1) ■ EOWPVT 85.2 (14.8) / 83.9 (14.3) ■ PPVT-4 90.8 (14.6) / 91 (15.) ■ SS/CBS 16.5 (0.2) / 17.9 (0.2) ■ SS/CBS/PALS 12.2 (0.1) / 12.6 (0.1) ■ WJ-III-AP 93.6 (14.7) / 91.2 (15.2) ■ WJ-III-LWI 104.4 (19.1) / 99.3 (14.4) ■ WJ-III-S 97.5 (14) / 97.4 (14.6)	■ **Statistics Extracted:** B, SE, Effect Size ■ **Covariates:** child/family level—pretest scores, child age at assessment, gender, ethnicity, language, household poverty ratio, maternal education, maternal depressive symptoms, time interval between the fall and spring assessments; program level—SES, percent DLLs, percent using curriculum and assessment from the same package, teacher turnover, program mean salary
Bulotsky-Shearer 2014[38]^m^	■ **Publication**: Journal (EED) ■ **Design**: Cross-Sectional ■ **Dataset:** Head Start ■ **Country**: United States ■ **Sample size:** class 53; child, range by analyses 299–304 ■ **% Female:** 49 ■ **Mean age:** 47.8 ■ **Ethnicity:** African American 45%, Hispanic 44%, Caucasian 6%, Other 5% ■ **Mean maternal education**: 50% **Mean household income**: at least 200% below poverty	■ Classroom Organizations 4.59 (0.99) ■ Emotional Support 5.28(0.97) ■ Instructional Support (Literacy Focus not included) 2.48(1.05)	■ WJ-AP 391.20(26.70) ■ WJ-LWI 333.60(24.26) ■ WJ-PV 453.99(14.49)	■ **Statistics Extracted:** Pearson's Correlation, B, df, T-Test ■ **Covariates:** age, gender, ethnicity, caregiver age, caregiver marital status, caregiver education, language, play interaction, play disruption, play disconnection, interaction terms
Burchinal 2008[31]^A^	■ **Publication**: Journal (ADS) ■ **Design**: Cross-Sectional ■ **Dataset:** NCEDL (Multi-State & SWEEP) ■ **Country**: United States ■ **Sample size:** class 227; child, range by analyses 642–743 ■ **% Female:** 49.5 ■ **Mean age:** 54.2 mo. ■ **Ethnicity:** C42%, B21%, H23%, A4%, M9% ■ **Mean maternal education**: 12.42 years ■ **Mean household income**: $30,000	■ Instructional Climate (Concept Development and Quality of Feedback) 2.22 (0.78)	■ Acad. Rat. Scale 2.13 (0.82) ■ OWLS-Oral Exp. 91.46 (12.16) ■ PPVT-III 93.15 (13.71) ■ TCRS-SC 3.44 (0.74) ■ TCRS-PB 1.5 (0.51) ■ WJ-III-AP 97.6 (13.03)	■ **Statistics Extracted:** Pearson’s Correlation ■ **Covariates:** none
Burchinal 2010[39]^A^	■ **Publication**: Journal (ECRQ) ■ **Design**: Longitudinal ■ **Dataset:** NCEDL (Multi-State & SWEEP) ■ **Country**: United States ■ **Sample size:** class 671; child 1129 ■ **% Female:** 51 ■ **Mean age:** NR ■ **Ethnicity:** C12%, B21%, H36%, A3%, O1% ■ **Mean maternal education**: 11.7 years ■ **Mean household income**: NR	■ Emotional Climate (all Emotional Support dimensions and Behaviors Management dimension) 5.49 (0.68) ■ Instructional Climate (Concept Development and Quality of Feedback) 2.04 (0.85)	■ OWLS-Oral Exp. 90.39 (12.08) ■ PPVT-III 91.98 (13.4) ■ TCRS-SC 3.57 (0.79) ■ TCRS-PB 1.54 (0.57) ■ WJ-III-AP 408.83 (18.39) ■ WJ-III-LWI 337.13 (24.92)	■ **Statistics Extracted:** B, SE, T-Test ■ **Covariates:** child/family level—pretest score, gender, ethnicity, maternal education; classroom level—Head Start, public school
Burchinal 2011[40]: NCEDL Sample^m^^,^ ^A^	■ **Publication**: Chapter ■ **Design**: Longitudinal ■ **Dataset:** NCEDL (Multi-State & SWEEP) ■ **Country**: United States ■ **Sample size:** class 50+; child 1465 ■ **% Female:** 51 ■ **Mean age:** 60.6 mo. ■ **Ethnicity:** C29%, B21%, H36%, O15% ■ **Mean maternal education**: 11.8 years ■ **Mean household income**: NR	■ CLASS—Total Score (Emotional Climate and Instructional Climate) 4.5 (0.6) ■ Emotional Climate (all Emotional Support dimensions and Behaviors Management dimension) 5.5 (0.7) ■ Instructional Climate (Composite of Concept Development and Quality of Feedback) 2.1 (0.8)	■ PPVT-R 92 (13.4) ■ TCRS-SS 3.6 (0.8) ■ TCRS-PB 1.5 (0.6) ■ WJ-III-LWI 99.5 (13) ■ WJ-III-AP 96 (12.2)	■ **Statistics Extracted:** Partial Correlation, B, SE ■ **Covariates:** gender, ethnicity, maternal education, site
Burchinal 2012[41]^;^ Whole Sample 2012^m^^,^ ^A^; Sample B: Spanish-English Testing^A^; Sample C: Spanish-Spanish Testing^A^	■ **Publication**: Journal (ECRQ) ■ **Design**: Longitudinal ■ **Dataset:** NCEDL (Multi-State & SWEEP) ■ **Country**: United States ■ **Sample size:** ■ **Whole Sample**: class NR; child, range by analyses 199–301; **Sample A**: class NR; 58–84; **Sample B**: class NR; child, 193–267 ■ **% Female:** Whole Sample: 46; A: 38; B: 49 ■ **Mean age:** Whole Sample A, & B: about 48 mo. ■ **Ethnicity:** 100% Spanish-speaking ■ **Mean maternal education**: 2.82 on a 9-point scale (3 = High School or GED) ■ **Mean household income**: NR	■ **Whole Sample** ■ Emotional Climate (all Emotional Support dimensions and Behaviors Management dimension) 5.55 (0.58) ■ Instructional Climate (Composite of Concept Development and Quality of Feedback) 1.97 (0.86)	■ PPVT-III 80.35 (11.17) ■ TVIP 84.17 (14.43) ■ WJ-III-LWI 92.26 (12.26) ■ WJ-III-AP 92.94 (11.94) ■ WM-LWI 91.01 (13.07) ■ WM-AP 9.89 (14.72)	■ **Statistics Extracted:** Pearson's Correlation, B, SE, Cohen's d ■ **Covariates:** pretest score, gender, maternal education, state, analysis group, English literacy screener (PreLAS), no instruction in Spanish, percent of instruction in Spanish, interaction terms
Burchinal 2014[42]^m^	■ **Publication**: Journal (ECRQ) ■ **Design**: Longitudinal ■ **Dataset:** Family Life Project ■ **Country**: United States ■ **Sample size:** class NR; child 822 ■ **% Female:** 49 ■ **Mean age:** NR ■ **Ethnicity:** African-American 43%, Caucasian 57%, Other <1% ■ **Mean maternal education**: 15.38 ■ **Mean household income**: 2.05	■ Classroom Organization 4.82(0.86) ■ Emotional Support 5.35(0.70) ■ Instructional Support (Literacy Focus not included) 2.60(0.95)	■ Behaviors Problems -0.01 (0.77) ■ Behavioral Competence 0.01 (0.76) ■ PPVT-III 94.03 (16.04) ■ TOPEL 92.98 (14.65) ■ WJ-LWI 99.53 (12.44) WJ-AP 100.71 (12.58) ■ Working Memory Task 0.32 (0.68)	■ **Statistics Extracted:** Pearson's Correlation, B, SE, F-Ratio ■ **Covariates:** pretest score, state, ethnicity, gender, maternal education, marital status, income/needs ratio, H.O.M.E. score, hours in care
Curby 2013[43]^m^^,^^A^	■ **Publication**: Journal (EED) ■ **Design:** Longitudinal ■ **Dataset:** NCEDL (Multi-State & SWEEP) ■ **Country**: United States ■ **Sample size:** class 655; child, range by analyses 1758–2439 ■ **% Female:** 51 ■ **Mean age:** 4.62 years ■ **Ethnicity:** NR ■ **Mean maternal education**: 12.90 ■ **Mean household income**: 52.5 below 150% of the poverty for family size	■ CLASS (9 scale ver.) ■ Emotional Support 5.74 (.69)	■ Letter Naming 13.89 (9.43) ■ PPVT- III 96.30 (14.32) ■ OWLS 93.64 (13.02) ■ TCRS Competence 3.56 (.77) ■ TCRS Problem Behaviors (1.57 (.61 ■ WJ-III-R 3.65 (4.02) ■ WJ-III-AP 99.11 (12.85)	■ **Statistics:** Pearson's Correlation, B, df, T-Test ■ **Covariates:** pretest score, gender, ethnicity, poverty, maternal education, emotional support consistency
Dang 2011[44]; NCEDL Sample^A^	■ **Publication**: Report ■ **Design**: Longitudinal ■ **Dataset:** NCEDL (Multi-State & SWEEP) ■ **Country**: United States ■ **Sample size:** class 721; child 2982 ■ **% Female:** 51 ■ **Mean age:** 55.5 mo. ■ **Ethnicity:** C41%, B18%, H22%, O14% ■ **Mean maternal education**: NR ■ **Mean household income**: NR	■ Instructional Climate (Composite of Concept Development and Quality of Feedback) 2.06 (0.84)	■ PPVT-III 52.25 (18.2) ■ WJ-III-AP 99.11 (12.85)	■ **Statistics Extracted:** B, SE ■ **Covariates:** pretest, gender, child’s age, ethnicity, maternal education, age at baseline assessment, family income at or below 150 percent of poverty line, number of people in household, household (a) grandma present, (b) father present, (c) step-father present, ECERS-R, interaction terms
Dobbs-Oates 2011[45]^m^^,^^X^	■ **Publication**: Journal (ECRQ) ■ **Design**: Longitudinal ■ **Country**: United States ■ **Sample size:** class 67; child, range by analyses about 1/3 of 398 and 398 ■ **% Female:** 51 ■ **Mean age:** 52 mo. ■ **Ethnicity:** C41%, B39%, H6%, O14% ■ **Mean maternal education**: NR ■ **Mean household income**: NR	■ Teacher Behaviors Management 4.81 (1.09)	■ PALS-PreK-Alphabet 17.66 (8.96) ■ PPVT-III 95.22 (13.82) ■ PWPA-Alphabet 108.77 (19.46) ■ PWPA/PALS Comp 0.04 (0.84)	■ **Statistics Extracted:** B, SE ■ **Covariates:** child/family level—pretest score, age, ethnicity, children’s task orientation; classroom level—intervention condition
Dominguez 2010[46]	■ **Publication**: Journal (SPR) ■ **Design**: Longitudinal ■ **Country**: United States ■ **Sample size:** class 29; child 275 ■ **% Female:** 51 ■ **Mean age:** 53.6 mo. ■ **Ethnicity:** C40%, H56%, O4% ■ **Mean maternal education**: NR ■ **Mean household income**: NR	■ Classroom Organization 4.76 (0.9) ■ Emotional Support 5.41 (0.80) ■ Instructional Support 2.74 (0.61)	■ Galileo System 573.59 (44.92)	■ **Statistics Extracted:** B, T-Test ■ **Covariates:** pretest score, age at start, shyness
Dotterer 2012[47]^m^^,^^A^	■ **Publication**: Journal (ECDC) ■ **Design**: Longitudinal ■ **Dataset:** NCEDL (Multi-State & SWEEP) ■ **Country**: United States ■ **Sample size:** class 716; child 3584 ■ **% Female:** 51.17 ■ **Mean age:** 48 mo. ■ **Ethnicity:** C41%, B18%, H27%, O14% ■ **Mean maternal education**: 12.62 years ■ **Mean household income**: $36,041	■ Emotional Climate (all Emotional Support dimensions and Behaviors Management dimension) 5.65 (0.68) ■ Instructional Climate (Composite of Concept Development and Quality of Feedback) 2.06 (0.82)	■ Acad. Rat. Scale 92.22 (0.93) ■ Naming Letters 6.2 (3.65) ■ Naming Numbers 11.71 (9.33) ■ OWLS-Exp. Lang 90.61 (12.24) ■ PPVT-III 92.22 (13.32) ■ WJ-III-Rhyming 2.76 (3.43) ■ WJ-III-AP 96.11 (12.26)	■ **Statistics Extracted:** B, SE ■ **Covariates:** child/family level—gender, ethnicity, maternal education; classroom level—hours per day, % Caucasian, poverty, program, poverty x program, teacher education, staff-child ratio, ECERS—Language & Interaction, ECERS—Provision for Learning
Downer 2012[48]; Sample A: Dual Language Learners^m^^,^ ^A^; Sample B: Latino^A^	■ **Publication**: Journal (ECRQ) ■ **Design**: Longitudinal ■ **Dataset:** NCEDL (Multi-State & SWEEP) ■ **Country**: United States ■ **Sample size:** class 721; child **Sample A** 956, **Sample B** 328 ■ **% Female:** 51 ■ **Mean age:** NR ■ **Ethnicity:** C40%, B18%, H26%, O16% ■ **Mean maternal education**: 12.6 years ■ **Mean household income**: NR (over 58% of families were at or below 150% of the federal poverty threshold)	■ **Whole Sample** ■ CLASS (9 scale ver.) ■ Classroom Organization 0.0 (0.86) ■ Emotional Support 0.0 (0.47) ■ Instructional Support (Language Modeling and Literacy Focus not included) 0.01 (0.84)	■ **Whole Sample** ■ ARS-Language 3.0 (0.97) ■ TCRS-SS 0.77 (3.64) ■ TCRS-PB 1.57 (0.75) ■ WJ-III-AP 412.19 (18.88) ■ WJ-III-LWI 12.9 (9.61)	■ **Statistics Extracted:** B, SE, T-Test ■ **Covariates:** child/family level—pretest score, gender, ethnicity, maternal education, poverty, language, test interval, test in Spanish, DLL status; classroom level—teacher education (BA), staff-child ratio, poverty, full-day, state, teacher/teacher’s assistant speaks Spanish, percent DLL, staff-child ratio, teacher education, interaction terms
Early 2006[30]^A^	■ **Publication**: Journal (ECRQ) ■ **Design**: Longitudinal ■ **Dataset:** NCEDL (Multi-State) ■ **Country**: United States ■ **Sample size:** class 237; child, range by analyses 804–809 ■ **% Female:** 51 ■ **Mean age:** 54.7 mo. ■ **Ethnicity:** C41%, B24%, H25%, A2%, M8% ■ **Mean maternal education**: NR ■ **Mean household income**: NR	■ Emotional (all Emotional Support dimensions and Behaviors Management dimension) 5.22 (0.76) ■ Instructional Climate (Composite of Concept Development and Quality of Feedback) 2.47 (1.10)	■ Identifying Colors 9.29 (1.73) ■ Identifying Letters 12.26 (9.5) ■ Identifying Numbers 6.26 (3.67) ■ OWLS-Oral Exp. 94.79 (12.29) ■ PPVT-III 95.69 (13.58) ■ WJ-III-AP 98.56 (11.86) ■ WJ-III-SA 2.95 (3.54)	■ **Statistics Extracted:** Pearson's Correlation ■ **Covariates:** state, fall scores, program in school, hours per week, maternal education, staff-child ratio, teacher education, ECERS
Gosse 2014[49]^m^	■ **Publication**: Journal (EED) ■ **Design:** Longitudinal ■ **Dataset:** Sample Pianta et al., 2008 ■ **Country**: United States ■ **Sample size:** class 95; child 360 ■ **% Female:** 49 ■ **Mean age:** 53 mo. ■ **Ethnicity:** B45%. C28%, H7%, Multiracial7%, A6%, O3% ■ **2014 Mean maternal education**: NR ■ **Mean household income**: NR	■ Instructional Support (Literacy Focus not included) 3.1 (.5)	■ NAP-SF 19.5 (6.7)	■ **Statistics:** B, SE ■ **Covariates:** pretest score, age, gender, ethnicity, maternal education, English at home, fall literacy composite, study condition, RS-closeness, RS-conflict, interaction terms
Guo 2010[50]^A^	■ **Publication**: Journal (TEACH &TEACH ED) ■ **Design**: Longitudinal ■ **Country**: United States ■ **Dataset:** NCEDL (Multi-State & SWEEP) ■ **Sample size:** class 67; child 322 ■ **% Female:** 49 ■ **Mean age:** 48 mo. ■ **Ethnicity:** C42%, B40%, H6%, A1%, M11% ■ **Mean maternal education**: NR ■ **Mean household income**: NR	■ Emotional Support 4.69 (1.07) ■ Instructional Support (Literacy Focus not included) 3.45 (0.97)	■ PPVT-III 58.31 (17.24) ■ Print Awareness 0.04 (0.84)	■ **Statistics Extracted:** B, SE, T-Test ■ **Covariates:** pretest score, age, gender, ethnicity, maternal education, English at home, fall literacy composite, treatment condition, interaction terms
Guo 2014[51]^m^^,^^U^	■ **Publication**: Journal (EED) ■ **Design:** Longitudinal ■ **Dataset:** PCER ■ **Country**: United States ■ **Sample size:** class 16; child 130 ■ **% Female:** 45 ■ **Mean age:** 53.76 mo. ■ **Ethnicity:** C72%, B21%, H4% ■ **2014 Mean maternal education**: NR ■ **Mean household income**: 38,062	■ Teacher Behaviors Management 5.67 (.81)	■ PPVT-III 68.94 (15.68)	■ **Statistics Extracted:** B, SE ■ **Covariates:** child/family level—pretest score, age, gender, family income, classroom age SD, interaction terms
Hamre 2010[52]	■ **Publication**: Journal (ECRQ) ■ **Design**: Longitudinal ■ **Country**: United States ■ **Sample size:** class 154; child 680 ■ **% Female:** 51 ■ **Mean age:** NR ■ **Ethnicity:** NR ■ **Mean maternal education**: 12.8 years ■ **Mean household income**: NR	■ Total Score (Regard for Student Perspectives, Language Modeling, Literacy Focus not included) 4.43 (0.56) ■ Language Modeling 2.73 (0.71) ■ Literary Focus 2.46 (0.86)	■ Pre-CTOPP-Total 59.4 (12.6) ■ Pre-CTOPP-Phonological 30.6 (5.3) ■ Pre-CTOPP-Print Awareness 28.6 (7.5) ■ Pre-CTOPP-Receptive Vocab. 33.7 (3.5)	■ **Statistics Extracted:** B, SE, Effect Size ■ **Covariates:** child/family level—pretest score, days between testing, days absent, gender, non-English, maternal education; classroom level–percent non-English, mean mother's education, dosage, adherence, teacher (a) education, (b) field of study, (c) ECE), (d) years teaching pre-K
Hamre 2014[53]^m^	■ **Publication**: Journal (CD) ■ **Design:** Longitudinal ■ **Dataset:** Sample Hamre et al., 2012 ■ **Country**: United States ■ **Sample size:** class 314; child 1407 ■ **% Female:** 51 ■ **Mean age:** 4.17 years ■ **Ethnicity:** B47%, H34%, C11.4%, A2.4%, O5.2% ■ **2014 Mean maternal education**: NR (generally low) ■ **Mean household income**: NR	■ Emotional Support 5.11 (0.87) ■ Classroom Organization 5.05 (0.76) ■ Instructional Support (Literacy dimension not included) 2.36 (0.86)	■ Backward Digit Spin- 1.35 (.69) ■ Pencil Tap .64 (.33) ■ PPVT-III 50.59 (19.51) ■ STRS-Closeness 4.49 (.58) ■ STRS-Conflict 1.80 (.92) ■ TOPEL-PA 14.88 (5.57) ■ TOPEL-PK 21.42 (11.32) ■ WJ-PV 13.61 (3.66)	■ **Statistics Extracted:** B, SE ■ **Covariates:** child/family level—pretest score, age, gender, ethnicity, days between assessments, maternal education, intervention group; classroom level—teacher education, teacher experience, income to needs, Head Start, Public School, curriculum
Hestenes 2015[54]^m^	■ **Publication**: Journal (ECRQ) ■ **Design**: Cross-sectional ■ **Country**: United States ■ **Sample size:** class 97; child 422 ■ **% Female:** 52 ■ **Mean age:** 48.5 mo. ■ **Ethnicity:** B29%, C58%, H5% ■ **Mean maternal education**: NR ■ **Mean household income**: NR	■ Emotional Support 5.3 (.82) ■ Classroom Organization 4.43 (.96) ■ Instructional Support 1.64 (.53)	■ SSIS—Internalizing Problems ■ SSIS—Externalizing Problems ■ SSIS—Learning Self-Efficacy ■ SSIS—Social Skills	■ **Statistics Extracted:** B, SE ■ **Covariates:** child’s age, gender, hours per week at center
Howes 2008[7]^A^	■ **Publication**: Journal (ECRQ) ■ **Design**: Longitudinal ■ **Dataset:** NCEDL (Multi-State & SWEEP) ■ **Country**: United States ■ **Sample size:** class 701; child, range by analyses 1787–2044 ■ **% Female:** 51 ■ **Mean age:** NR ■ **Ethnicity:** C42%, O58% ■ **Mean maternal education**: 12.8 years ■ **Mean household income**: NR	■ Emotional Climate (all Emotional Support dimensions and Behaviors Management dimension) NR ■ Instructional Climate (Composite of Concept Development and Quality of Feedback) NR	■ ARS—Language/Literacy NR ■ Identifying Letters NR ■ OWLS-Oral Exp. NR (NR) ■ PPVT-R NR ■ SSPBS-SS NR ■ SSPBS-BP NR ■ WJ-III-AP NR	■ **Statistics Extracted:** Pearson’s Correlation, B, SE ■ **Covariates:** child/family level—state, gender, child age at fall assessment, ethnicity, maternal education, poverty, number of people in the household; classroom level—teacher education (BA), ratios, in/out school, full/part-day, T-C relationship, ECERS-R Provisions for Learning for learning, ECERS-R Interactions
Johnson 2013[55]	■ **Publication**: Journal (ECRQ) ■ **Design**: Longitudinal ■ **Country**: United States ■ **Sample size:** class 28; child 177 ■ **% Female:** 43 ■ **Mean age:** 3.03 years to 5.18 years ■ **Ethnicity:** B60%, H36% ■ **Mean maternal education**: NR ■ **Mean household income**: 90% below poverty line	■ Emotional Support NR	■ ASBI 55.89 (7.49) ■ PPVT-III 87.35 (13.18)	■ **Statistics Extracted:** B, SE ■ **Covariates:** pretest scores, gender, ethnicity, age, intervention group, PPVT score, caregiver depression, interaction terms
Keys 2013[56]: NCEDL Sample^A^	■ **Publication**: Journal (CD) ■ **Design**: Longitudinal ■ **Dataset:** NCEDL (Multi-State & SWEEP) ■ **Country**: United States ■ **Sample size:** class 721; child 2982 ■ **% Female:** 51 ■ **Mean age:** 55.5 mo. ■ **Ethnicity:** C41%, B18%, H26%, O14% ■ **Mean maternal education**: NR ■ **Mean household income**: NR	■ Emotional Climate (all Emotional Support dimensions and Behaviors Management dimension) 5.56 (0.68) ■ Instructional Climate (Composite of Concept Development and Quality of Feedback) 2.07 (0.84)	■ PPVT-III 96.3 (14.31) ■ TCRS-PB 1.57 (0.7) ■ TCRS-SS Skills 3.64 (0.7) ■ WJ-III-AP 99.11 (12.85)	■ **Statistics Extracted:** B, SE ■ **Covariates:** child/family level—pretest scores, gender, ethnicity, school readiness skills (cognitive, attention, and externalizing Behaviors problems), maternal education, child’s age at fall and spring assessments, family income at or below 150% of poverty, no. of people in household, grandma present, father present, step father present, interaction terms
Logan 2011[57]; Sample A: State Funded; Sample B: Head Start^m^	■ **Publication**: Journal (CYCF) ■ **Design**: Longitudinal ■ **Country**: United States ■ **Sample size A**: class 14; child 129 ■ **Sample size B**: class 46; child 160 ■ **% Female: A** 50; **B**: 46 ■ **Mean age: A** 53.1mo. **B** 52.2mo. ■ **Ethnicity A:** C71%, B21%, H5%, O2% ■ **Ethnicity B:** C40%, B39%, H9%, O12% ■ **Mean maternal education A**: 1.33 on 3-point scale (1 = High School or GED) ■ **Mean maternal education B**: 2.28 on 6-point scale (0 = less than 8^th^ grade to 6 = doctoral degree) ■ **Mean household income A**: $26,550 ■ **Mean household income B**: NR	■ Sample A ■ Instructional Support (Literacy dimension not included) 3.55 (0.98) ■ Sample B ■ Instructional Support (Literacy dimension not included) 3.07 (1.07)	■ Sample A ■ MUBI 8.77 (3.03) ■ Sample B ■ NAP 14.77 (8.37)	■ **Statistics Extracted:** B, SE, df ■ **Covariates:** age, maternal education, household income, children’s attendance, interaction term
Mashburn 2009[58]^]^^A^	■ **Publication**: Journal (CD) ■ **Design**: Longitudinal ■ **Dataset:** NCEDL (Multi-State & SWEEP) ■ **Country**: United States ■ **Sample size:** class 453; child, range by analyses 1680–1681 ■ **% Female:** 51 ■ **Mean age:** NR ■ **Ethnicity:** C52%, B23%, H11%, O15% ■ **Mean maternal education**: 13.1 years ■ **Mean household income**: NR	■ Emotional Climate (all Emotional Support dimensions and Behaviors Management dimension) 5.56 (0.67)	■ OWLS-Oral Exp. 94.9 (12.7) ■ PPVT-III 97.9 (14.1)	■ **Statistics Extracted:** B, SE ■ **Covariates:** pretest score, sex, race, maternal education, peer language, full-day, class size, ratio
Mashburn, Pianta 2008[8]^A^	■ **Publication**: Journal (CD) ■ **Design**: Longitudinal ■ **Dataset:** NCEDL (Multi-State & SWEEP) ■ **Country**: United States ■ **Sample size:** class 671; child, range by analyses 2307–2439 ■ **% Female:** 51 ■ **Mean age:** NR ■ **Ethnicity:** C46%, B21%, H27%, O15% ■ **Mean maternal education**: 12.9 years ■ **Mean household income**: NR	■ Emotional Climate (all Emotional Support dimensions and Behaviors Management dimension) 5.56 (0.68) ■ Instructional Climate (Composite of Concept Development and Quality of Feedback) 2.08 (0.83)	■ Letter Naming 13.9 (9.42) ■ OWLS-Oral Exp. 93.6 (13) ■ PPVT-III 96.3 (14.3) ■ TCRS-SS 3.66 (0.7) ■ TCRS-PB 1.49 (0.54) ■ WJ-III-SA 3.65 (4.02) ■ WJ-III-AP 99.1 (12.)	■ **Statistics Extracted:** B, SE ■ **Covariates:** pretest scores, gender, ethnicity, mother’s education, poverty, state
McGinty 2012[59]^X^	■ **Publication**: Journal (ECRQ) ■ **Design**: Longitudinal ■ **Country**: United States ■ **Sample size:** class 59; child 379 ■ **% Female:** 54 ■ **Mean age:** 51.9 mo. ■ **Ethnicity:** C42%, B37%, O21% ■ **Mean maternal education**: NR ■ **Mean household income**: 56% earn <$30,000	■ CLASS Total Score 4.39 (0.9)	■ PALS Pre-K NR (NR) ■ PWPA-PALS (High/Low) 0.02 (NR) ■ PWPA-PALS(High/Mod) 0.04 (NR) ■ PWPA-PALS (High/High) 0.07 (NR) ■ PWPA-PALS (Low/Low) -0.34 (NR) ■ PWPA-PALS (Low/Mod) -0.16 (NR) ■ PWPA-PALS (Low/High) 0.02 ■ PWPA 8.59 (3.91)	■ **Statistics Extracted:** B, SE, df ■ **Covariates:** child level—pretest score, age, language ability, attentional skills; classroom level—literacy environment, explicit print instruction, intervention condition
Peisner-Feinberg 2008[60]^;^ Sample: NC More at Four (2007–08)^m^	■ **Publication**: Report ■ **Design**: Longitudinal ■ **Dataset:** NC More at Four (2007–08) ■ **Country**: United States ■ **Sample size:** class 50; child 321 ■ **% Female:** 54 ■ **Mean age:** 55.2 mo. ■ **Ethnicity:** C29%, B36%, H25%, A2%, O8% ■ **Mean maternal education**: NR ■ **Mean household income**: NR	■ Classroom Organization 5.3 (0.8) ■ Emotional Support 5.8 (0.9) ■ Instructional Support (Literacy dimension not included) 3.0 (0.9)	■ Counting Task 18 (11) ■ PPVT-4 91 (17.2) ■ Social Awareness 4.2 (1.6) ■ SSRS-SS 109.4 (14.6) ■ SSRS-PB 99.5 (13.2) ■ TOPEL-Print Knowledge 95.8 (14.1) ■ TOPEL-Phonological Awareness 85.3 (15.2) ■ WJ-III-LWI 96.5 (12.3) ■ WJ-III-AP 98.2 (12.3)	■ **Statistics Extracted:** T-Test ■ **Covariates:** pretest, gender, age at first assessment, days elapsed since previous assessment, days of attendance at More at Four
Peisner-Feinberg 2013[61]^m^	■ **Publication**: Report ■ **Design**: Longitudinal ■ **Dataset:** Georgia Pre-K (2011–12) ■ **Country**: United States ■ **Sample size:** class 99; child, range by analyses 454–469 ■ **% Female:** 52 ■ **Mean age:** 50.4 mo. ■ **Ethnicity:** C35%, B39%, H15%, A4%, M3%, O4% ■ **Mean maternal education**: NR ■ **Mean household income**: NR	■ Classroom Organization 5.2 (0.8) ■ Emotional Support 5.5 (0.8) ■ Instructional Support 2.8 (0.8)	■ Counting 11.7 (1.0) ■ Naming Letters 8.0 (0.0) ■ Social Awareness 4.8 (1.3) ■ SSIS-Prob. Behaviors 100.3 (15.8) ■ SSIS-Social Skills 100.2 (14.7)	■ **Statistics Extracted:** B, SE ■ **Covariates:** child/family level—gender, child’s age at fall assessment, family income, English proficiency; classroom level—program type (school, private), lead teacher certified, lead teacher years of experience teaching pre‐k, percent non-English‐speaking children in class, ECERS‐R Total score
Reid 2013[62]^A^	■ **Publication**: Journal (EED) ■ **Design:** Longitudinal ■ **Dataset:** NCEDL (Multi-State & SWEEP) ■ **Country**: United States ■ **Sample size:** class 704; child 2966 ■ **% Female:** NR ■ **Mean age:** NR ■ **Ethnicity:** NR ■ **2014 Mean maternal education**: 12.8 ■ **Mean household income**: 32, 574	■ CLASS (9 scale ver.) ■ Emotional Support ■ Low SES classrooms -0.26 (1.04) ■ Mid SES classrooms 0.02 (0.98) ■ High SES classrooms 0.42 (0.82) ■ Instructional Support (Language Modeling and Literacy Focus not included) ■ Low SES classrooms -0.05 (0.98) ■ Mid SES classrooms -0.02 (1.00) ■ High SES classrooms 0.12 (1.02)	■ Hightower NR ■ OWLS-Oral Exp. NR ■ PPVT NR ■ WJ- III AP NR	■ **Statistics Extracted:** Beta ■ **Covariates:** child/family level—pretest score, gender, age, SES, ethnicity, single parent, ELL status, IEP status; classroom level—SES, deviation of income, percent Caucasian, teacher has BA, teacher has more than a BA, class size (less than 18), full-day, Head Start, interaction terms
Sabol 2013[63]^A^	■ **Publication**: Journal (EED) ■ **Design**: Longitudinal ■ **Dataset:** NCEDL (Multi-State & SWEEP) ■ **Country**: United States ■ **Sample size:** class 673; child 2419 ■ **% Female:** NR ■ **Mean age:** 4.61 ■ **Ethnicity:** C42%, B25%, H18%, O15% ■ **Mean maternal education**: 12.96 years ■ **Mean household income**: NR	■ CLASS (9 scale ver.) ■ Total Score Language Modeling and Literacy dimension not included) 4.46 (0.62)	■ Letter Knowledge 14.40 (9.34) ■ OWLS-Oral Exp. 93.21 (13.45) ■ PPVT- III 95.52 (14.70) ■ Pre-reading 3.36 (3.82) ■ TCRS-SS 3.56 (0.77) ■ TCRS-PB 1.49 (0.55) ■ WJ-III AP 98.88 (13.37)	■ **Statistics Extracted:** Pearson’s Correlation, Beta ■ **Covariates:** child/family level—pretest score, gender, ethnicity, maternal education, poverty, household size, attend pre-k prior year; classroom level -state, ethnicity, Head Start
Weiland 2013[64]^m^	■ **Publication**: Journal (ECRQ) ■ **Design**: Longitudinal ■ **Country**: United States ■ **Sample size:** class 83; child 414 ■ **% Female:** 50.0 ■ **Mean age:** 26.33 mo. ■ **Ethnicity:** C16%, B28%, H43%, A11% ■ **Mean maternal education**: NA ■ **Mean household income**: NA	■ Classroom Organization 5.10 (0.68) ■ Emotional Support 5.63 (0.60) ■ Instructional Support 4.30 (0.84)	■ BDS 1.44 (0.72) ■ FDS 4.51 (1.25) ■ PPVT-III 94.45 (17.89) ■ Pencil Tap 11.65 (5.35)	■ **Statistics:** B, SE ■ **Covariates:** child/family level—pretest scores, ethnicity, gender, home language, free/reduced lunch, special needs, age, attendance zone, pre-BPS experience; classroom level—teacher education (MA), group size
West 2010[65]^m^^,^^B^ (2^nd^ doc. Malone 2010[66])	■ **Publication**: Report ■ **Design**: Longitudinal ■ **Dataset:** FACES 2006 ■ **Country**: United States ■ **Sample size:** class 410; child, range by analyses 426–684 ■ **% Female:** 49 ■ **Mean age:** 36–48 mo. ■ **Ethnicity:** C25%, B27%, H39%, A2%, M5%, O3% ■ **Mean maternal education**: NR ■**Mean household income**: NR	■ Instructional Support NR	■ ECLS-Math 19 (NR) ■ PPVT-4 95 (NR) ■ SSRS-BP 6.7 (NR) ■ SSRS-SS 18 (NR) ■ WJ-III-LWI 334.5 (NR) ■ WJ-III-AP 401.5 (NR)	■ **Statistics Extracted:** Beta ■ **Covariates:** child/family level—child's exposure to HS (1 vs. 2 years), gender, ethnicity, language, poverty ratio, joint book reading at least 3 times per week, number of books in home, maternal education, parent depressive symptoms, Low/mid/high ability at HS entry; classroom level—mean peer abilities at HS entry on WJ-AP, variation in peer abilities at HS entry on WJ-AP, full day/half day, ECERS—Teaching and Interactions, ECERS—Provisions for Learning for Learning, teacher education (Has a BA)
Xu 2014[67]	■ **Publication**: Journal (ECE) ■ **Design**: Cross-Sectional ■ **Dataset:** Head Start ■ **Country**: United States ■ **Sample size:** class 14, child 248 (analyses at classroom level) ■ **% Female:** NA ■ **Mean age:** 3 year olds (34%) and 4 year olds (66%) ■ **Ethnicity:** B98%, H4% ■ **Mean maternal education**: NR ■**Mean household income**: NR	■ Classroom Organization NR ■ Emotional Support NR ■ Instructional Support NR	■ PPVT-IV 90.75 (13.67) ■ PALS-PreK 5.21 (2.03) ■ Lower-case Recognition 21.22 (4.29) ■ TOPEL-PK 100.73 (13.91) ■ TOPEL-DV 95.50 (11.89) ■ TOPEL-PA 90.47(12.88)	■ **Statistics Extracted:** T-Test, Beta ■ **Covariates:** age

Abbreviations: NR = Not Reported; C = Caucasian, B = African American, H = Hispanic, A = Asian, M = Mixed, O = Other. For all other acronyms, please refer to S5 File.

^a^Descriptives provided reflect characteristics (actual or estimates) of the sample/research design for which data was extracted for the current study and therefore may represent a subsample/analysis of the larger study.

^b^This paper is one of a series of “Meta-Analyses and Systematic Reviews” assessing the relationship between child care quality and children’s outcomes; therefore, uppercase superscript letters below are in reference to various large databases that samples in these papers were drawn from. These letters have been kept consistent across the series of papers for our readers.

^c^CLASS was operationalized in a number of different ways.

^d^Scale of measurement for the means and standard reported in this table varied across studies (e.g., percentiles, standard scores, raw score). All outcomes used in the current paper are presented in S3 File.

^e^All covariates used in the described sample are listed, but may vary by analyses.

^m^Studies included in the meta analyses

^**A**^National Center for Early Development and Learning Dataset (NCEDL)

^**B**^Head Start Family and children Experiences Survey (FACES) 2006 Cohort

^**M**^Head Start Family and children Experiences Survey (FACES, 2009) Cohort

^**U**^Preschool Curriculum Evaluation Research (PCER, 1999–2003)

^**X**^Ohio Virginia (2005–2006, 2006–2007)

### Outcomes

In an effort to include the many outcomes that have been associated with high quality “instruction” for children [15] we defined “outcomes” broadly. We included any measure of children’s academic competence (e.g., cognitive, language, mathematics) as well as health/wellbeing and social and emotional functioning.

### Meta-Analyses

Studies that could be meta-analyzed were drawn from the pool of studies that were eligible for the systematic review. To be meta-analyzed studies had to use identical child outcome measures and had to use an identical operationalization of the CLASS. We adopted a minimum requirement of three studies to conduct a meta-analysis on a particular child outcome. To increase homogeneity among studies that were meta-analyzed, only studies that ensured children’s exposure to the program were included. To do this, we only included studies in which the authors explicitly stated that children had been in the program for a period of time prior to their assessment. We also included analyses in which children’s pre-scores were used as a covariate, or in which gain scores were used in analyses because such analyses required that children have spent a period of time in the program. In addition, only statistics that accounted for covariates (e.g., child and family characteristics) were combined within a single meta-analysis. Finally, to insure independence of samples, when multiple papers drew from the same dataset, only the study with the largest sample size was selected for inclusion in meta-analyses. Thus, within a given meta-analysis, only one coefficient from each sample was included in any one analysis.

Statistical models with quadratic terms assume non-linear associations between the variables. Given the statistics extracted for most studies only tested for linear relationships (correlation coefficients and linear regression coefficients), associations in models using quadratic terms were excluded and only results examining linear relationships alone were used in the meta-analyses. We used random-effects models for meta-analyses. All meta-analyses were conducted using the software Comprehensive Meta-Analysis Version 3 (See S3 File) [68]. We used the *I*^*2*^ statistic [69] as an indicator of statistical heterogeneity across studies. Low *I*^*2*^ values suggest homogeneity between studies.

### Assessment of Risk of Biases Among Included Studies

We planned to assess risk of bias in included studies using Newcastle-Ottawa scale [22,70]. However, initial review revealed that because of the correlational nature of research in this area, the standard questions on this checklist did not enable us to discriminate differences in study quality in our sample of studies. All included studies had moderate to high risk of bias due to issues with sample selection, exposure assessment and confounder adjustment. However, the inclusion criteria we used for the meta-analysis selected for the better quality studies. This means that the studies in the meta-analyses used well-researched, standardized measures, they tended to use covariates in their analyses and to ensure that the children in these studies had at least some exposure to the program before exploring whether program quality was associated with their outcomes. Finally, the majority of the papers we included were published in peer-reviewed journals.

## Results

### Search Results

**Fig 1:**
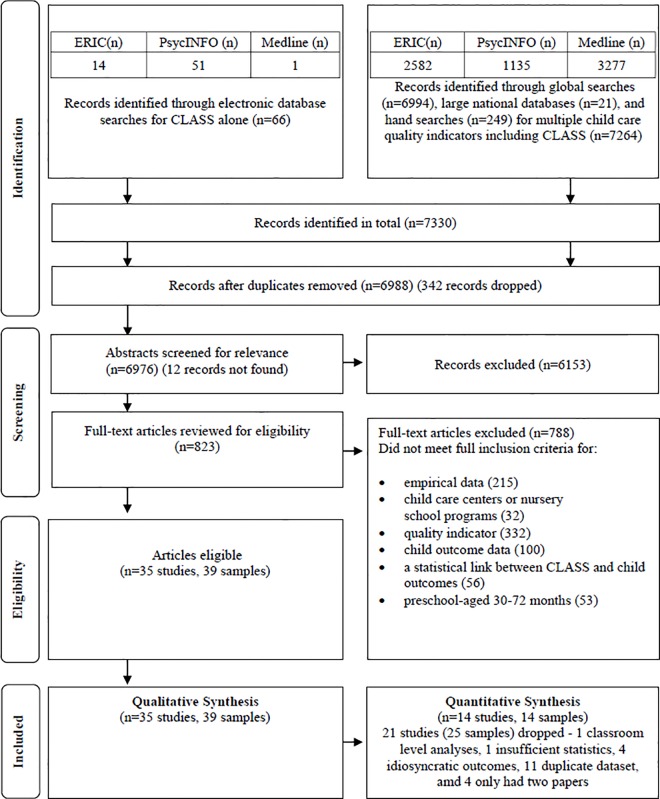
Flow diagram for study selection. Adapted from Moher, 2009 [71].

Details of the search and study selection are provided in Fig 1. Thirty five studies were included in this review (consisting of 39 samples) [7,8,30,31,34,36,38–63], which included 28 journal articles, 6 reports and 1 book chapter.

### Description of Studies

The characteristics of the 35 eligible studies are described Table 2 [7,8,30,31,34,36,38–63]. All of the studies were conducted in the USA. Sixteen studies [7,8,30,31,39–41,43,44,47,48,50,56,58,62,63] came from the NCEDL database and two studies from the FACES 2006 project. Also, two studies used data collected in Ohio and Virginia between 2005 and 2007 [45,59]. Thirty-one studies [7,8,30,34,36,39–53,55–65] were longitudinal and 4 studies [31,38,54,67] were cross-sectional.

Of the independent samples, (i.e., excluding overlapping datasets by retaining only the largest sample size), the total sample size was 15,167 preschool-aged children. Sample sizes ranged from 129 to 3584 children (Median = 398). All of the studies had similar numbers of boys and girls (46% to 62% males). All but one study [58] sampled at-risk children, with 47% to 100% of the children coming from low-income families. Children were primarily Caucasian, Hispanic, or Black.

### Operationalization of the CLASS

Studies used the CLASS dimensions, domains or total score. Nineteen studies [34,36,38,42,45,46,49–51,53–55,57,59–61,64,65,67] used the most recent version of the CLASS [72], 5 studies [43,48,52,62,63] used an earlier version [73], which did not include the Language Modeling dimension, and 11 studies [7,8,30,31,39–41,44,47,56,58] used CLASS domains (emotional and/or instructional climate) from the earlier version. Since we did not have data on the comparability of scores when these scales were combined vs. analyzed separately we chose the conservative route and tested the Emotional Climate domain separately. In terms of dimensions available for inclusions in this review, 3 studies [34,36,52] examined Language Modeling, 2 [45,51] examined Behavior Management, 1 study [36] examined Positive Climate and 1 study [52] examined Literacy Focus.

### Outcomes

As noted above, outcomes were grouped into cognitive (for which Backward Digit was the most frequently used measure), language (where Peabody Picture Vocabulary Test, PPVT; Woodcock-Johnson Letter-Word Identification, WJ-LWI and the Oral and Written Language Scales, OWLS, are used most often), mathematics (where the Woodcock-Johnson Applied Problems, WJ-AP and Early Childhood Longitudinal Study Birth Cohort Math, ECLS-B math, are used most frequently) and social/emotional outcomes (where the Social Skills Rating System, SSRS, and Teacher Child Relationship Scale, TCRS, are used most often). The 55 different child outcomes identified through this process are listed in S2 File.

### Systematic Review

**Fig 2:**
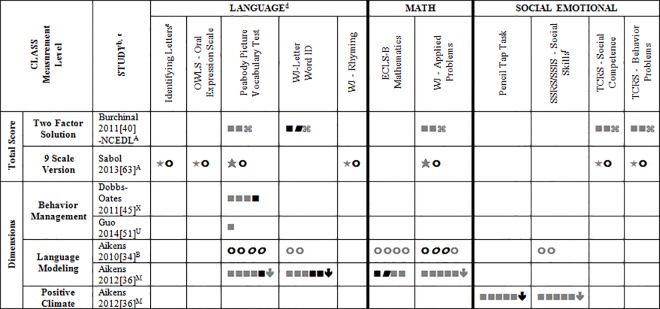
Systematic review of associations between the CLASS Total Score or Dimension and child outcomes. ^a^ Abbreviations: Symbols bolded are significant and positive, symbols bolded and italicized are significant and negative, and symbols in grey are non-significant. Star = Zero Order Pearson’s Correlation, Unfilled circle = Beta, Filled square = Unstandardized Coefficient, Black diamond minus white X = T-Test, Key clover = Partial Correlation, Downward arrow = Effect Size, Filled circle = F-Ratio. Total Score (Two Factor Solution) = Total Score for Emotional Climate and Instructional Climate; Total Score (9 Scale Version) = Total Score with Language Modeling and Literacy Focus dimension not included. For more details, see Table 2 in this manuscript. ^a^To improve the readability of this complex table, six papers [46,49,52,55,57,59] that had an outcome that appeared in only one or two samples were omitted from this figure. Several analyses from other papers that had idiosyncratic outcomes were also excluded. For a comprehensive display of all of the data for all of the child outcomes see Tables A-D, S4 File. ^b^This paper is one of a series of Meta-Analyses and Systematic Reviews assessing the relationship between child care quality and children’s outcomes; therefore, superscript letters below are in reference to various large databases that samples in these papers were drawn from. These letters have been kept consistent across the series of papers for our readers. ^c^Samples within papers are described in more detail in Table 2 in this manuscript. ^d^Acronyms for child outcomes are listed in S5 File. ^e^Identifying Letters (also referred to as Letter Knowledge, Letter-Naming, Naming Letters).^f^SSRS/SSIS problem behaviour also includes individual scales: internalizing and externalizing for Hestenes et al., 2015 [54].^A^National Center for Early Development and Learning Dataset (NCEDL); ^B^Head Start Family and Children Experiences Survey (FACES 2006 Cohort); ^M^Head Start Family and Children Experiences Survey (FACES, 2009 Cohort); ^U^Preschool Curriculum Evaluation Research (PCER, 1999–2003);^X^Ohio Virginia (2005–2006, 2006–2007).

**Fig 3:**
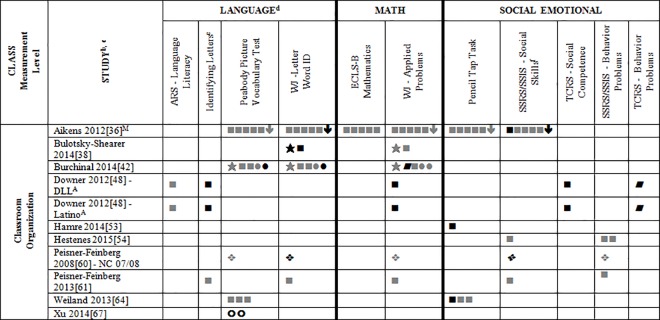
Systematic review of associations between the CLASS domain of Classroom Organization and child outcomes. ^a^ Abbreviations: Symbols bolded are significant and positive, symbols bolded and italicized are significant and negative, and symbols in grey are non-significant. Star = Zero Order Pearson's Correlation, Unfilled circle = Beta, Filled square = Unstandardized Coefficient, Black diamond minus white X = T-Test, Key clover = Partial Correlation, Downward arrow = Effect Size, Filled circle = F-Ratio. ^a^To improve the readability of this complex table, six papers [46,49,52,55,57,59] that had an outcome that appeared in only one or two samples were omitted from this figure. Several analyses from other papers that had idiosyncratic outcomes were also excluded. For a comprehensive display of all of the data for all of the child outcomes see Tables A-D, S4 File.^b^This paper is one of a series of Meta-Analyses and Systematic Reviews assessing the relationship between child care quality and children’s outcomes; therefore, superscript letters below are in reference to various large databases that samples in these papers were drawn from. These letters have been kept consistent across the series of papers for our readers. ^c^Samples within papers are described in more detail in Table 2 in this manuscript. ^d^Acronyms for child outcomes are listed in S5 File. ^e^Identifying Letters (also referred to as Letter Knowledge, Letter-Naming, Naming Letters).^f^SSRS/SSIS problem behaviour also includes individual scales: internalizing and externalizing for Hestenes et al., 2015 [54].^A^National Center for Early Development and Learning Dataset (NCEDL); ^M^Head Start Family and Children Experiences Survey (FACES, 2009 Cohort).

**Fig 4:**
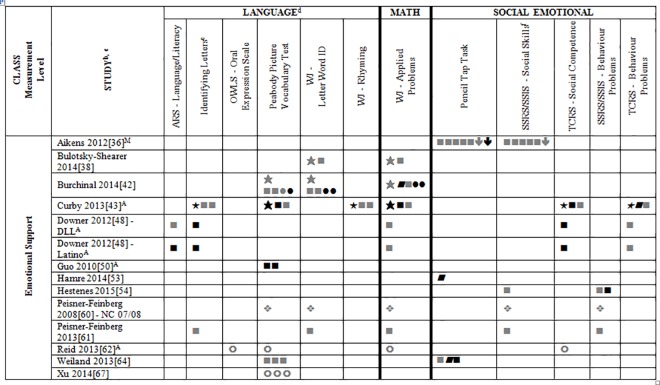
Systematic review of associations between the CLASS domain of Emotional Support and child outcomes. ^a^ Abbreviations: Symbols bolded are significant and positive, symbols bolded and italicized are significant and negative, and symbols in grey are non-significant. Star = Zero Order Pearson’s Correlation, Unfilled circle = Beta, Filled square = Unstandardized Coefficient, Black diamond minus white X = T-Test, Key clover = Partial Correlation, Downward arrow = Effect Size, Filled circle = F-Ratio. ^**a**^To improve the readability of this complex table, six papers [46,49,52,55,57,59] that had an outcome that appeared in only one or two samples were omitted from this figure. Several analyses from other papers that had idiosyncratic outcomes were also excluded. For a comprehensive display of all of the data for all of the child outcomes see Tables A-D, S4 File. ^**b**^This paper is one of a series of Meta-Analyses and Systematic Reviews assessing the relationship between child care quality and children’s outcomes; therefore, superscript letters below are in reference to various large databases that samples in these papers were drawn from. These letters have been kept consistent across the series of papers for our readers. ^**c**^Samples within papers are described in more detail in Table 2 in this manuscript. ^d^Acronyms for child outcomes are listed in S5 File. ^**e**^Identifying Letters (also referred to as Letter Knowledge, Letter-Naming, Naming Letters). ^f^SSRS/SSIS problem behaviour also includes individual scales: internalizing and externalizing for Hestenes et al., 2015 [54].^A^National Center for Early Development and Learning Dataset (NCEDL); ^M^Head Start Family and Children Experiences Survey (FACES, 2009 Cohort).

**Fig 5:**
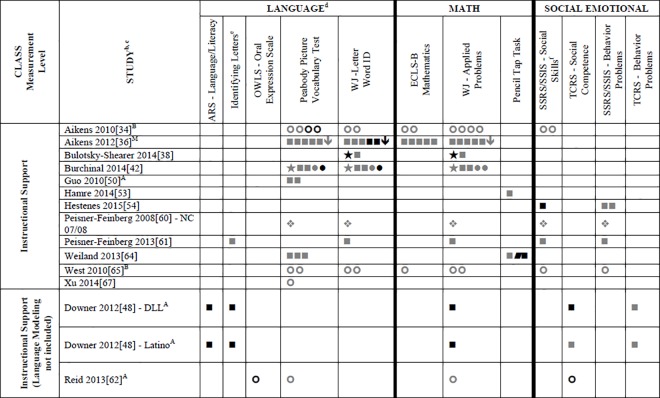
Systematic review of associations between the CLASS domain of Instructional Support and child outcomes. ^a^ Abbreviations: Symbols bolded are significant and positive, symbols bolded and italicized are significant and negative, and symbols in grey are non-significant. Star = Zero Order Pearson’s Correlation, Unfilled circle = Beta, Filled square = Unstandardized Coefficient, Black diamond minus white X = T-Test, Key clover = Partial Correlation, Downward arrow = Effect Size, Filled circle = F-Ratio. ^**a**^To improve the readability of this complex table, six papers [46,49,52,55,57,59] that had an outcome that appeared in only one or two samples were omitted from this figure. Several analyses from other papers that had idiosyncratic outcomes were also excluded. For a comprehensive display of all of the data for all of the child outcomes see Tables A-D, S4 File. ^**b**^This paper is one of a series of Meta-Analyses and Systematic Reviews assessing the relationship between child care quality and children’s outcomes; therefore, superscript letters below are in reference to various large databases that samples in these papers were drawn from. These letters have been kept consistent across the series of papers for our readers. ^**c**^Samples within papers are described in more detail in Table 2 in this manuscript. ^d^Acronyms for child outcomes are listed in S5 File. ^**e**^Identifying Letters (also referred to as Letter Knowledge, Letter-Naming, Naming Letters).^f^SSRS/SSIS problem behaviour also includes individual scales: internalizing and externalizing for Hestenes et al., 2015 [54].^A^National Center for Early Development and Learning Dataset (NCEDL); ^B^Head Start Family and Children Experiences Survey (FACES 2006 Cohort); ^M^Head Start Family and Children Experiences Survey (FACES, 2009 Cohort).

**Fig 6:**
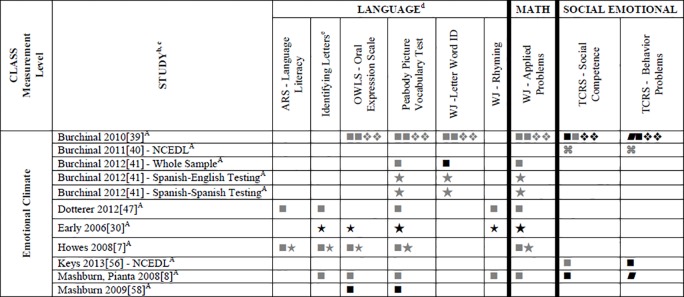
Systematic review of associations between the CLASS domain of Emotional Climate and child outcomes. ^a^ Abbreviations: Symbols bolded are significant and positive, symbols bolded and italicized are significant and negative, and symbols in grey are non-significant. Star = Zero Order Pearson’s Correlation, Unfilled circle = Beta, Filled square = Unstandardized Coefficient, Black diamond minus white X = T-Test, Key clover = Partial Correlation, Downward arrow = Effect Size, Filled circle = F-Ratio. ^**a**^To improve the readability of this complex table, six papers [46,49,52,55,57,59] that had an outcome that appeared in only one or two samples were omitted from this figure. Several analyses from other papers that had idiosyncratic outcomes were also excluded. For a comprehensive display of all of the data for all of the child outcomes see Tables A-D, S4 File. ^**b**^This paper is one of a series of Meta-Analyses and Systematic Reviews assessing the relationship between child care quality and children’s outcomes; therefore, superscript letters below are in reference to various large databases that samples in these papers were drawn from. These letters have been kept consistent across the series of papers for our readers. ^**c**^Samples within papers are described in more detail in Table 2 in this manuscript. ^d^Acronyms for child outcomes are listed in S5 File. ^**e**^Identifying Letters (also referred to as Letter Knowledge, Letter-Naming, Naming Letters). ^A^National Center for Early Development and Learning Dataset (NCEDL).

**Fig 7:**
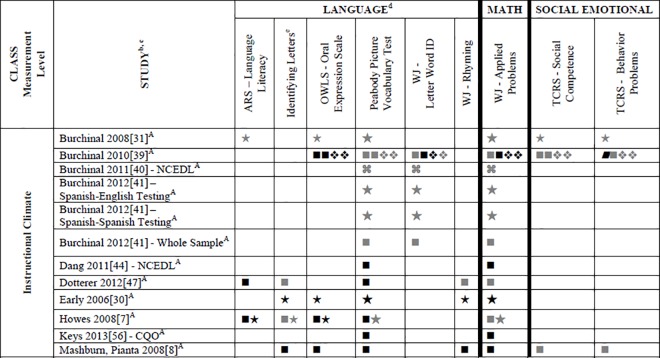
Systematic review of associations between the CLASS domain of Instructional Climate and child outcomes. ^a^ Abbreviations: Symbols bolded are significant and positive, symbols bolded and italicized are significant and negative, and symbols in grey are non-significant. Star = Zero Order Pearson’s Correlation, Unfilled circle = Beta, Filled square = Unstandardized Coefficient, Black diamond minus white X = T-Test, Key clover = Partial Correlation, Downward arrow = Effect Size, Filled circle = F-Ratio. ^**a**^To improve the readability of this complex table, six papers [46,49,52,55,57,59] that had an outcome that appeared in only one or two samples were omitted from this figure. Several analyses from other papers that had idiosyncratic outcomes were also excluded. For a comprehensive display of all of the data for all of the child outcomes see Tables A-D, S4 File. ^**b**^This paper is one of a series of Meta-Analyses and Systematic Reviews assessing the relationship between child care quality and children’s outcomes; therefore, superscript letters below are in reference to various large databases that samples in these papers were drawn from. These letters have been kept consistent across the series of papers for our readers. ^**c**^Samples within papers are described in more detail in Table 2 in this manuscript. ^d^Acronyms for child outcomes are listed in S5 File. ^**e**^Identifying Letters (also referred to as Letter Knowledge, Letter-Naming, Naming Letters). ^A^National Center for Early Development and Learning Dataset (NCEDL).

Data for 55 different child outcome measures (see Table 2 where all studies are described and S2 File where all outcomes are listed) were reported in included studies. All of this data (which is presented in Tables A-D, S4 File) was included in the systematic review. To simplify this heterogeneity and help with visual interpretation, child outcomes that were reported in 3 or more samples are reported in Figs 2–7. Of the 35 eligible articles, 29 studies [7,8,30,31,34,36,38–45,47,48,50,51,53,54,56,58,60–65,67] provided data for at least one outcome variable that was also included in at least two other samples. Six studies [46,49,52,55,57,59] are not included in Figs 2–7 because they report only outcomes found in less than three samples.

Figs 2–7 show relationships between domains and dimensions of the CLASS and outcomes in individual studies. Data is presented for 5 language outcomes, 3 mathematics outcomes and 5 social/emotional outcomes. For language outcomes, a high number of significant associations for the OWLS, PPVT, WJ Letter Word ID (and letter identification measures that were not part of the WJ) and WJ Rhyming were identified in the included studies. However, these results mostly came from studies using NCEDL database. Few significant associations remained when the studies that used the NCEDL [7,8,30,31,39–41,43,44,47,48,50,56,58,62,63] dataset were excluded. Results from studies that used NCEDL data included in this systematic review should be interpreted with caution as the samples in these publications are drawn from a single dataset and therefore overlap. There were a few positive associations between CLASS scores and the math outcome of WJ Applied Problems but none with the ECLS-B Mathematics measure. Few positive significant associations were reported between the various CLASS domains and dimensions and measures of child outcomes.

We also explored whether any one domain or dimension of the CLASS had a stronger pattern of associations with any of our outcomes. We paid particular attention to CLASS domains and dimensions that were conceptually linked to specific child outcomes (e.g., Emotional Support to children’s social outcomes, Instructional Support for Learning to language and mathematics or Language Modeling to language outcomes). Across domains and dimensions there were few associations identified and most of them used from NCEDL data. Overall a consistent lack of relationships between the CLASS and the various outcomes reported in the literature was observed.

We also explored whether accounting for covariates influenced the pattern of results. We did this by comparing results in studies that provided both a Pearson’s correlation and either partial correlations, betas or unstandardized regression coefficients. Five studies [7,38,42,43,63] presented these statistics for the same operationalization of the CLASS and child outcome. Findings across these five studies were highly consistent indicating that when covariates were added there were fewer significant associations compared to the Pearson’s correlations. The one exception to this was Sabol, 2013 [63] in which the significance level of most of the Pearson correlations was not reported. As a final check, we compared studies that used overlapping samples and found that results were highly consistent among them.

### Meta-Analyses

**Fig 8:**
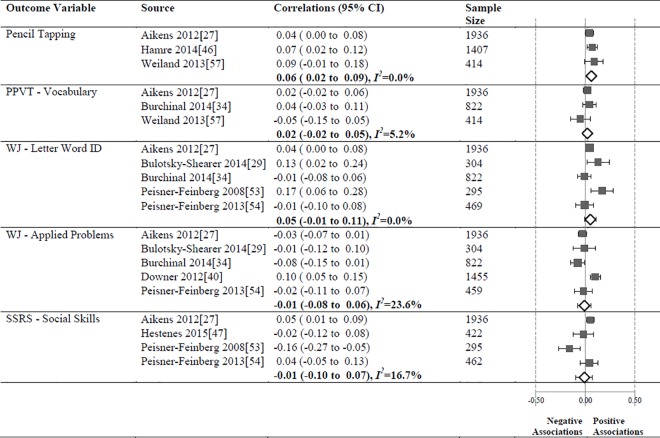
Meta-analyses of associations between the CLASS domain of Classroom Organization and child outcomes.

**Fig 9:**
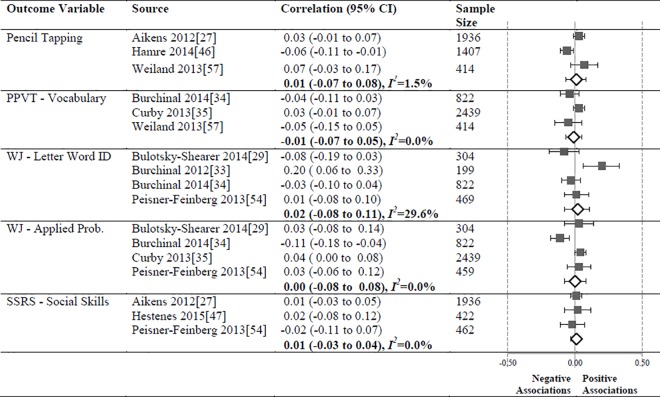
Meta-analyses of associations between the CLASS domain of Emotional Support and child outcomes.

**Fig 10:**
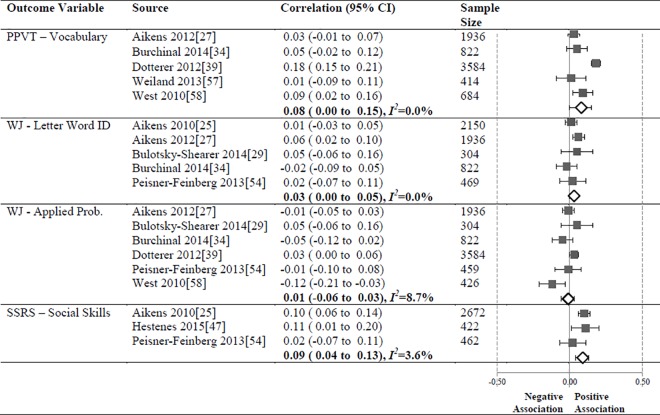
Meta-analyses of associations between the CLASS domain of Instructional Support and child outcomes.

When samples overlapped across studies we retained only the study with the largest sample. Thus, 10 studies [7,8,30,31,39,44,50,56,58,62] from the NCEDL database were dropped. We also dropped 1 study [46] with insufficient statistics, 1 study [67] with classroom level analyses, and 4 studies [52,55,59,63] with idiosyncratic outcomes. This left a total of 19 independent samples [34,36,38,40–43,45,47–49,51,53,54,57,60,61,64,65] that provided 28 unique relationships of a particular operationalization of CLASS and a child outcome that meet our criteria for the meta-analyses (See Figs 8–10).

#### Classroom Organization

The meta-analysis (Fig 8) revealed significant but small correlations between the Classroom Organization domain and Pencil Tapping (N = 3757, pooled correlation coefficient 0.06; 95% CI: 0.04 to 0.09, *I*^*2*^ = 0%).). The relationships between the Classroom Organization domain and PPVT Vocabulary (N = 3172), WJ LWI (N = 3826), WJ Applied Problems (N = 4976), and SSRS Social Skills (N = 3115) subscales were not significant.

#### Emotional Support

The meta-analysis (Fig 9) revealed no significant associations between the Emotional Support domain and Pencil Taping (N = 3757), PPVT Vocabulary (N = 3675), WJ Letter Word ID (N = 1794), WJ Applied Problems (N = 4024), and SSRS Social Skills (N = 2820).

#### Instructional Support

The meta-analysis (Fig 10) revealed significant but small correlation between the Instructional Support domain the SSRS Social Skills subscale (N = 3556, 0.09; 95% CI: 0.02 to 0.12, *I*^*2*^ = 3.6%). The relationships between the Instructional Support domain and PPVT (N = 7740) and WJ Letter Word ID (N = 7146) were not significant. The relationship between Instructional Support and the WJ Applied Problems (N = 7531) was also not significant.

## Discussion

The CLASS is a relatively new measure of ECEC quality that focuses on constructs that are supported by a long history of theory (e.g., attachment theory, ecological systems theory, etc.) and research on the kids of environments that support child development. The field has embraced the measure as evidenced by its growing use in Quality Ratings and Improvement Systems in the US. One of the appeals of the CLASS is that the measure developers have paid close attention to testing its psychometric properties. Thus, despite being a relatively new measure we found 35 studies that could be used in this review. The quick uptake of this measure in research studies allows us to draw some conclusions about the extent to which it is associated with child outcomes.

In this systematic review and meta-analyses, we found a limited number of significant associations between the CLASS and the language social outcomes available in the literature. This was especially true when the large number of studies that used the NCEDL data was accounted for. Meta-analyses of studies that used the same operationalization of the CLASS, had identical outcome measures and had statistics that could be converted to *r* revealed some relationships between the different domains of the CLASS and child outcomes. Of the 14 meta-analyses we conducted, 2 showed significant, although small effect sizes. The low *I*^*2*^ values found across these analyses suggest that the studies within each meta-analysis were homogeneous and support our conservative approach of only meta-analyzing studies that were quite similar to one another in terms of operationalization of the CLASS and child outcome.

The lack of relationships is especially surprising in cases in which the CLASS domains maps onto a specific child outcome measure most closely (e.g., Emotional Support and children’s Social Skills measured using the SSRS). However, because the studies we were able to meta-analyze were limited, we did not have an opportunity to test some of these relationships (e.g., we were not able to meta-analyze the relationship between language modeling and children’s vocabulary). Our finding that associations between the CLASS and child outcomes are limited likely reflects the multitude of family and other factors the impact the outcomes we explored in this paper. Below we discuss some of the methodological limitations of the research we covered. We then provide alternative explanations for the weak associations.

The literature in this area is problematic because: 1) it is heterogeneous and 2) many studies have methodological limitations. First, potential for selection bias in the included studies in this review is non-trivial as a number of studies did not randomly select programs and many programs and parents decline to participate. For example, 16 studies [7,8,30,31,39–41,43,44,47,48,50,56,58,62,63] included in this review used data from the NCEDL database. The NCEDL is a large-scale study that draws on randomly selected programs from a number of diverse states. While this study is clearly an important one, it consists of a single sample. Approximately 78% of randomly selected programs in this study agreed to participate while 55–61% of parents from these programs agreed for their children to take part in the study. Thus, even in this relatively methodologically strong study, the final sample may not be representative of the general population of children. Moreover, sample self-selection may contribute to the low variability in CLASS scores observed. With few exceptions, the standard deviations reported for the CLASS domains and dimensions were smaller than 1. This self-selection may result in an over estimate of the quality of existing programs as better programs may be more willing to participate in research on program quality. It may be possible to achieve higher response rates in studies that are part of government initiatives to improve regulation of ECEC programs. Thus, researchers and government or other agencies involved in oversight of ECEC programs need to work together towards improving the quality of research in this area. In general parents who agree to participate in research tend to be better educated. This parent self-selection likely skews the types of children who participate in published studies. Another limitation of the samples used in these CLASS studies is that they all come from the United States. Clearly more research is needed to explore the role of the process quality, as captured in the CLASS, in other jurisdictions.

Second, scores on the domains and dimensions of the CLASS; though consistent across programs, were not always favorable. Scores for Instructional Support were consistently low with values at around “2” whereas Classroom Organization and Emotional Support scores were higher at around “5” on this 7-point scale. This indicates that Instructional Support is an area in particular need of quality improvement across programs. The low scores observed for some domains and dimensions are especially worrisome given our speculation that these are likely to be overestimates of quality due to sample self-section issues.

Finally, there was significant heterogeneity in the outcomes included in these studies. There are two versions of the CLASS, some researchers reported a total score, others created their own profiles from different CLASS codes. To maximize the number of studies we could draw from we attempted to “streamline” the CLASS scales as much as possible by equating domains that were made up of very similar dimensions and including studies that provided data on some dimensions only. There was also substantial variability in the statistics that were reported which further limited our ability to meta-analyze findings across papers. Finally, there is enormous variability in the covariates used in different studies. For example, some studies reported zero order correlations using no covariates [31], while other studies reported only partial correlations or regressions that included anywhere between 5 [39] and 22 [34] covariates controlling for child, family and program factors in their analyses [32]. Clearly studies in this area need to be conducted and reported more consistently so that findings can be integrated more effectively in the future.

Studies about the effects of child care generally have some methodological limitations. For example, for ethical as well as logistical reasons, it is not possible to randomly assign children to programs of differing quality. As a result, all of the studies we included are observational/correlational and causal conclusions about the link between the CLASS and child outcomes cannot be drawn. Reporting of study methodologies and results is inconsistent and at times incomplete. For example, a measure of variability was not reported in a number of studies [7,55,65–67]. However, it is worth noting that the studies included in our meta-analyses used psychometrically strong measurements, had large sample sizes, included many covariates in the analyses in an effort to mitigate the program and parent self-selection issues discussed above, and ensured that children who were assessed had been exposed to the programs. Thus, while the studies we included suffered from substantial limitations, they also had many strengths.

One major drawback of this heterogeneity and the number of studies available for meta-analysis is that we could not test the effects of possible moderators on the relationship between the CLASS and child outcomes. Variables of particular interest are whether the duration of exposure and socioeconomic backgrounds of children influence associations between the CLASS and child outcomes. In general, ECEC programs are expected to make the greatest impact on children from low SES backgrounds. While we were not able to test SES as a moderator, the studies we included generally over represented lower income children. Future reviews based on additional studies to be published in the future in this area, will hopefully be able to test these issues more directly.

Other explanations for the small effect sizes found in this review include the possibility that higher quality programs only impact children who require especially supportive environments. The differential susceptibility hypothesis [74] posits that children who are more vulnerable for genetic, temperamental or other reasons, may be differentially impacted by the extent to which their surroundings are supportive. Thus, overall effects may be eliminated/reduced when aggregating across diverse children within a given sample. Another possible explanation is that the CLASS measures quality at the classroom level, while classrooms in the early childhood sector are staffed by multiple adults. Measures like the Caregiver Interaction Scale [75], which are collected at the staff level, are worth exploring. However, these generally focus only on warmth and sensitivity without the emphasis on developmentally appropriate instruction offered by the CLASS. Discrepancies in the unit of analysis may be contributing to the lack of associations reported in this review. In addition, it is possible that other aspects of interaction should be attended to. For example, Hamre et al. (2013) [14] mention the “use of emotion words and emotion coaching” as “unique elements of classroom interactions not measured by the CLASS” (p. 464). Alternatively, child outcomes might be better predicted by more comprehensive measures such as the ECERS-R described earlier, that capture both structural and process quality. In addition, while we set out to collect a broad range of child outcomes, we could only report on outcomes that were found in the literature. Some outcomes (e.g., gross and fine motor abilities) are notably absent and could explain the lack of associations observed in this study.

Future studies should look at the presence of non-linear relationships between quality characteristics and child outcomes, as there may be some threshold value in ECEC quality that changes the direction of the association. Such relationships cannot be adequately captured by statistical indices that assume linear relationships reported here. The possibility that there are thresholds of quality in terms of impacts on children is gaining attention from researchers. This is illustrated by the recent publication of a special issue on this topic [76]. However, one study that tested this possibility directly with the CLASS did not provide support for the threshold hypothesis for this measure [77]. Finally, one of the strengths of this study is that we cast a very wide net in terms of our initial searches. This resulted in many “hits” that were not, in the end, relevant to the goals of this study. We then developed and systematically applied a set of selection criteria with the aim of having a relatively homogenous sample of studies for subsequent analysis. The downside of this approach is that it was very labour intensive. The upside is that we can be more confident that our searches were comprehensive.

As described earlier, the CLASS is being used increasingly as part of quality assurance programs. It is also used as an integral part of professional development (PD) programs where with the help of a coach, caregivers use the CLASS as a lens through which staff co-view videos of their own interactions with children to improve their practice. The logic of such PD programs stems from the rich literature that formed the basis for approaches like Teaching Through Interaction and informed the development of the CLASS. Yet, given the results of the current systematic review and meta-analysis, clearly more research about what aspects of the classroom environment are associated with child outcomes is needed.

### Conclusion

The CLASS operationalizes difficult constructs such as scaffolding and contingent responding by ECEC staff, constructs that are thought to be key in supporting children’s development. The CLASS domain that was most closely linked to child outcomes was Instructional Support. Sadly, it is also, by far, the lowest scoring domain across studies. Very few effects were found for Classroom Organization and Emotional Support. However, it is important to note that based on these findings we cannot conclude that programs of lower or higher quality than what we found in the literature would not impact children. As noted above, it may well be that there are threshold/levels of quality that must be met for quality to impact children. One of the most important conclusions of this study is that more research, using stronger methodologies and a variety of samples is needed. Research with samples from outside the US is especially important. However, despite all of the methodological limitations discussed above, there is a growing body of research about associations between the CLASS and child outcomes and it is important to systematically review the existing literature about this influential measure. Unfortunately, based the existing literature, it appears that associations between the CLASS and child outcomes are quite limited.

## Supporting Information

S1 File Search Syntax CLASS, Tables A-D.
(PDF)

S2 File Child Outcomes CLASS. (PDF)

S3 File Formulas CLASS. (PDF)

S4 File Systematic Review Tables CLASS, Tables A-D.
(PDF)

S5 File Acronyms CLASS. (PDF)

S6 File PRISMA Checklist CLASS. (PDF)

S7 File Database CLASS. (ZIP)
