# Supplementary material for: A Systematic Review and Meta-Analysis of a Measure of Staff/Child Interaction Quality (the Classroom Assessment Scoring System) in Early Childhood Education and Care Settings and Child Outcomes
Source: PLoS One. 2016 Dec 30;11(12):e0167660. doi: 10.1371/journal.pone.0167660 (PMC5201239; doi:10.1371/journal.pone.0167660)
Supplement: S2 File — (PDF) [file pone.0167660.s002.pdf]

A Systematic Review and Meta-Analysis of a Measure of Staff/Child Interaction Quality (the Classroom Assessment Scoring System) in Early Childhood Education and Care Settings and Child Outcomes

Supplemental Information 2

List of Child Outcome Variables

Outcome Category	Number of Measures	Child Outcome Variable	Number of Studies	Number of Samples
Approach	n=1	Galileo System for the Electronic Management of Learning - Language/Literacy	1	1
Cognitive	n=5	Backwards Digit Span (Carlson, 2005)[1]	2	2
		Backwards Digit Span (Gathercole & Pickering, 2000)[2]		
		FACES - Social Awareness Task	2	2
		Forward Digit Span	1	1
		Identifying Colors	1	1
		Working Memory Task	1	1
Language	n=26	Academic Rating Scale: Language & Literacy	4	5
		Expressive One Word Picture Vocabulary Test (English - EOWPVT)	1	1
		Expressive One Word Picture Vocabulary Test (Spanish – EOWPVT-SBE)		
		Identifying Letters (Letter Knowledge, Letter-Naming, Letter naming Test, Naming Letters)	8	9
		Language (PPVT and WJ Picture Vocabulary)	1	1
		MUBI	1	1
		Narrative Assessment Protocol (NAP)	2	2
		Narrative Assessment Protocol-SF (12 items)		
		Oral & Written Language Scales - Oral Expression Scale (OWLS)	8	8
		Oral & Written Language Scales - Total Scale (OWLS)	3	3
		PALS Pre-K – Lower Case Recognition (PALS Pre-K)	1	1
		PALS Pre-K - Total mean score (PALS Pre-K)	1	1
		Peabody Picture Vocabulary Test (English, PPVT-4)	25	27
		Peabody Picture Vocabulary Test - III (PPVT)		
		Test de Vocabulario en Imagenes Peabody (Spanish, TVIP)		
		Pre-CTOPP - Phonological Awareness (Pre-CTOPP)	1	1
		Pre-CTOPP - Print Awareness (Pre-CTOPP)	1	1
		Pre-CTOPP - Receptive Vocabulary (Pre-CTOPP)	1	1
		Print Awareness: Composite score Preschool Word and Print Awareness test (PWPA) and two Phonological Awareness Literacy Screening: Pre-Kindergarten (PALS) measures: Alphabet Knowledge subtest, Name Writing Subtest	2	2
		Print Knowledge: Composite score Preschool Word and Print Awareness test (PWPA) and one Phonological Awareness Literacy Screening: Pre-Kindergarten	1	1

A Systematic Review and Meta-Analysis of a Measure of Staff/Child Interaction Quality (the Classroom Assessment Scoring System) in Early Childhood Education and Care Settings and Child Outcomes

List of Child Outcome Variables				
**Outcome Category**	**Number of Measures**	**Child Outcome Variable**	**Number of Studies**	**Number of Samples**
		(PALS) measures: Upper Case Alphabet Knowledge subtest		
		TOPEL - Phonological Awareness	2	2
		TOPEL - Print Knowledge	1	1
		TOPEL - (Phonological Awareness + Print Knowledge)	1	1
		TOPEL - Total Score (Print Knowledge, Definitional Vocabulary, and Phonological Awareness)	1	1
		Woodcock Johnson - Letter Word Identification III (English, WJ LWI)	10	12
		Woodcock Johnson - Letter Word Identification (Spanish, WM LWI)		
		Woodcock Johnson - Picture Vocabulary III (WJ, English)	2	2
		Woodcock Johnson - Picture Vocabulary (WJ, Spanish)		
		Woodcock Johnson - Rhyming (English, WJ)	5	5
		Woodcock Johnson - Rhyming (Spanish, WM)		
		Woodcock Johnson - Sound Awareness (WJ)	1	1
		Woodcock Johnson - Spelling (English, WJ)	1	1
		Woodcock Johnson - Spelling (Spanish, WM)		
		Woodcock Johnson - Word Attack (WJ)	1	1
**Math**	n=4	Counting Task	2	2
		Early Childhood Longitudinal Study-Birth Cohort - Math (ECLS-B)	3	3
		Identifying Numbers (Naming Numbers)	2	2
		Woodcock Johnson - Applied Problems III (English, WJ AP)	21	24
		Bateria psico-educativa revisada de woodcock-Munoz - Revisada- Applied Problems (Spanish, WM AP)		
**Positive Behaviour**	n=10	Adaptive Social Behaviour Inventory - Prosocial Behaviour (express & comply) (ASBI)	1	1
		Early Childhood Longitudinal Study-Kindergarten Cohort - Approaches to Learning (ECLS-K)	1	1
		Learning Self-Efficacy	1	1
		Executive Functioning Pencil Tapping Task	3	3
		Social Skills (author created)	1	1
		Social Skills Improvement System - Social Skills (SSIS)	2	2
		Social Skills and Problem Behaviour Scale - Social Competence (SSPBS)	1	1
		Social Skills Rating System - Social Skills (Teacher, SSRS)	4	4
		Student Teacher Rating Scale – Closeness (TRS)	1	1

A Systematic Review and Meta-Analysis of a Measure of Staff/Child Interaction Quality (the Classroom Assessment Scoring System) in Early Childhood Education and Care Settings and Child Outcomes

**List of Child Outcome Variables**				
**Outcome Category**	**Number of Measures**	**Child Outcome Variable**	**Number of Studies**	**Number of Samples**
		Teacher Child Rating Scale - Social Competence (TCRS)	10	11
**Problem Behaviour**	n=9	Behaviour Problems Composite	2	2
		Behaviour Problems Composite	1	1
		Social Skills Improvement System - Externalizing Problems (SSIS)	1	1
		Social Skills Improvement System - Internalizing Problems (SSIS)	1	1
		Social Skills Improvement System - Problem Behaviours (SSIS)	1	1
		Social Skills and Problem Behaviour Scale – Problem Behaviours (SSPBS)	1	1
		Social Skills Rating System - Problem Behaviours (SSRS)	2	2
		Student Teacher Rating Scale – Conflict (TRS)	1	1
		Teacher Child Rating Scale - Behaviour Problems (TCRS)	8	9
**Total #**	55			
