# Supplementary material for: A Systematic Review and Meta-Analysis of a Measure of Staff/Child Interaction Quality (the Classroom Assessment Scoring System) in Early Childhood Education and Care Settings and Child Outcomes
Source: PLoS One. 2016 Dec 30;11(12):e0167660. doi: 10.1371/journal.pone.0167660 (PMC5201239; doi:10.1371/journal.pone.0167660)
Supplement: S3 File — (PDF) [file pone.0167660.s003.pdf]

A Systematic Review and Meta-Analysis of a Measure of Staff/Child Interaction Quality (the Classroom Assessment Scoring System) in Early Childhood Education and Care Settings and Child Outcomes

Supplemental Information 3
Formulas for Converting Statistics to r for Meta Analyses

Conversion to *t*, *d* or *r* through *t*-statistic

$$t = \frac{B}{SE(B)}$$

$$d = \frac{2t}{\sqrt{n-1}}$$

$$r = \frac{d}{\sqrt{d^2 + 4}}$$

where *B* is an unstandardized regression coefficient, *SE(B)* is its standard error, *d* is its standardized mean difference, *r* is the correlation effect size, and *n* is sample size.
