# Supplementary material for: A Systematic Review and Meta-Analysis of a Measure of Staff/Child Interaction Quality (the Classroom Assessment Scoring System) in Early Childhood Education and Care Settings and Child Outcomes
Source: PLoS One. 2016 Dec 30;11(12):e0167660. doi: 10.1371/journal.pone.0167660 (PMC5201239; doi:10.1371/journal.pone.0167660)
Supplement: S4 File — Systematic Review Tables CLASS, Tables A-D. (PDF) [file pone.0167660.s004.pdf]

A Systematic Review and Meta-Analysis of a Measure of Staff/Child Interaction Quality (the Classroom Assessment Scoring System) in Early Childhood Education and Care Settings and Child Outcomes

Supplemental Information 4
Systematic Review Results: All Outcomes

Table A. CLASS Predicting all Approach, Cognitive, and Math Outcomes

CLASS Measurement Level		STUDY[a, b]	APP[c]	COGNITIVE					MATH			
			Galileo	Backward Digit Span	FACES - Social Awareness Task	Forward Digit Span	Identifying Colours	Working Memory Task	Counting Task	ECLS-B Math	Identifying Numbers[d]	WJ Applied Problems
**Total Score**	**Factor Version**	Burchinal 2011[3] - NCEDL[A]										■■⌘
**Total Score**	**9 scale Version**	Sabol 2013[4,A]										★○
**CLASS Domains**	**Classroom Organization**	Aikens 2012[5,M]								■■■■■		■■■■■↓
		Bulotsky-Shearer 2014[6]										★■
		Burchinal 2014[7]						★■■●●				★■▰●●
		Dominguez 2010[8]	■■									
		Downer 2012[9] - DLL[A]										■
		Downer 2012[9] - Latino[A]										■
		Hamre 2014[10]		■								
		Peisner-Feinberg 2008[11] - NC 07/08			❖				❖			❖
		Peisner-Feinberg 2013[12]			■				■			■
		Weiland 2013[13]		■■■		■■■						
	**Emotional Support**	Bulotsky-Shearer 2014[6]										★■
		Burchinal 2014[7]						★■■●●				★▰■●●
		Curby 2013[14,A]										★■■
		Dominguez 2010[8]	■■									
		Downer 2012[9] - DLL[A]										■

A Systematic Review and Meta-Analysis of a Measure of Staff/Child Interaction Quality (the Classroom Assessment Scoring System) in Early Childhood Education and Care Settings and Child Outcomes

		Downer 2012[9] - Latino[A]										■
		Hamre 2014[10]		■								
		Peisner-Feinberg 2008[11] - NC 07/08			❖				❖			❖
		Peisner-Feinberg 2013[12]			■				■			■
		Reid 2013[15,A]										○
		Weiland 2013[13]		■■■		■■■						
	Instructional Support	Aikens 2010[16,B]								○○		○○○○
		Aikens 2012[5,M]								■■■■■		■■■■■↓
		Bulotsky-Shearer 2014[6]										★■
		Burchinal 2014[7]						★■■●●				★■■●●
		Dominguez 2010[8]	■■									
		Hamre 2014[10]		■								
		Peisner-Feinberg 2008[11] - NC 07/08			❖				❖			❖
		Peisner-Feinberg 2013[12]			■				■			■
		Weiland 2013[13]		■■■		■■■						
		West 2010[17,B]								○		○○
	Instructional Support (Language Modeling not included)	Downer 2012[9] - DLL[A]										■
		Downer 2012[9] - Latino[A]										■
		Reid 2013[15,A]										○
CLASS Factors	Emotional Climate	Burchinal 2010[18,A]										■■❖❖
		Burchinal 2012[19] - Whole Sample[A]										■
		Burchinal 2012[19] - Spanish-English Testing[A]										★
		Burchinal 2012[19] -Spanish-Spanish Testing[A]										★
		Dotterer 2012[20,A]									■	■
		Early 2006[21,A]					★				★	★
		Howes 2008[22,A]										■★
		Mashburn, Pianta 2008[23,A]										■
	Instructional Climate	Burchinal 2008[24,A]										★
		Burchinal 2010[18,A]										■■❖❖
		Burchinal 2011[3] - NCEDL[A]										⌘

		Burchinal 2012[19] - Whole Sample[A]										□
		Burchinal 2012[19] - Spanish-English Testing[A]										☆
		Burchinal 2012[19] - Spanish-Spanish Testing[A]										☆
		Dang 2011[25] - NCEDL[A]										■
		Dotterer 2012[20,A]									□	□
		Early 2006[21,A]					☆				★	★
		Howes 2008[22,A]										□☆
		Keys 2013[26] - NCEDL[A]										■
		Mashburn, Pianta 2008[23,A]										■
**Subscales**	**Language Modeling**	Aikens 2010[16,B]								○○○○		**O** *O* *O* ○
		Aikens 2012[5,M]								■ *■* □□		□□□□□⇩

Legend for Table

Significant & Positive	Significant & Negative	Nonsignificant	Statistic
★	*★*	☆	**r - Zero Order Pearson's Correlation**
**O**	*O*	○	**Beta**
■	*■*	□	**B (Unstandardized Coefficient)**
❖	*❖*	❖	**T-Test**
⌘	*⌘*	⌘	**Partial Correlation**
●	*●*	●	**F-Ratio**
⬇	*⬇*	⇩	**Effect Size**

Abbreviations: APP=Approaches to Learning; Total Score (factor ver.) = Total Score for Emotional Climate and Instructional Climate; Total Score (9 scale ver.)=Total Score with Language Modeling, Literacy Focus subscales not included. For more details please see Table 2 in the manuscript.

[a]This paper is one of a series of Meta-Analyses and Systematic Reviews assessing the relationship between child care quality and children's outcomes; therefore, superscript letters below are in reference to various large databases that samples in these papers were drawn from. These letters have been kept consistent across the series for our readers.

[b]Samples within papers are described in more detail in the manuscript in Table 2.

[c]Acronyms for child outcomes are listed in Supplemental Information 2.

[d]Identifying Numbers (also refers to as Naming Numbers).

[A]National Center for Early Development and Learning Dataset (NCEDL); [B]Head Start Family and Children Experiences Survey (FACES) 2006 Cohort; [M]Head Start Family and Children Experiences Survey (FACES, 2009) Cohort.

A Systematic Review and Meta-Analysis of a Measure of Staff/Child Interaction Quality (the Classroom Assessment Scoring System) in Early Childhood Education and Care Settings and Child Outcomes

Supplemental Information 4
Systematic Review Results: All Outcomes

Table B. CLASS Predicting all Language Outcomes A to P

CLASS Measurement Level		STUDY[a, b]	LANGUAGE[c]															
			ARS - Language & Literacy	Expressive One - Picture Vocabulary	Identifying Letters[d]	Language (PPVT+ WJ PV)	MUBI	Narrative Assessment Protocol	OWLS- Oral Expression Scale	OWLS- Total Scale	PALS Pre-K	PALS Pre-K - lower case recognition	PPVT - Vocabulary	Pre-CTOPP -Phonological Awareness	Pre-CTOPP -Print Awareness	Pre-CTOPP -Receptive Vocabulary	Print Awareness (PWPA and PALS Pre-K)	Print Knowledge (PWPA and PALS Pre-K)
**Total Score**	**Factor Version**	Burchinal 2011[3] - NCEDL[A]								⌘			■■<br>⌘					
	**11 Scale Version**	McGinty 2012[27,X]																■
	**9 Scale Version**	Sabol 2013[4,A]			○★				○★				○<br>★					
	**8 Scale Version**	Hamre 2010[28]									■			■	■	■		
**CLASS Domains**	**Classroom Organization**	Aikens 2012[5,M]		■■■<br>■■<br>↓									■■■<br>■■<br>↓					
		Burchinal 2014[7]											★					

													● ●(grey) ■(grey) ■(grey)					
		Downer 2012[9] - DLL[A]	■(grey)		■													
		Downer 2012[9] - Latino[A]	■(grey)		■													
		Hamre 2014[10]				■(grey)												
		Peisner-Feinberg 2008[11] - NC 07/08											❖(grey)					
		Peisner-Feinberg 2013[12]			■(grey)													
		Weiland 2013[13]											■■■(grey)					
		Xu 2014[29]										○○(grey)	○○					
		Burchinal 2014[7]											★(grey) ■■(grey) ● ●(grey)					
		Curby 2013[14,A]			■■(grey) ★					■■(grey) ★			■(grey) ■ ★					
		Downer 2012[9] - DLL[A]	■(grey)		■													
		Downer 2012[9] - Latino[A]	■		■													
	**Emotional Support**	Hamre 2014[10]				■(grey)												
		Guo 2010[30,A]											■■				■(grey) ■	
		Peisner-Feinberg 2008[11] - NC 07/08											❖(grey)					
		Peisner-Feinberg 2013[12]			■(grey)													
		Reid 2013[15]							○(grey)				○(grey)					
		Weiland 2013[13]											■■■(grey)					
		Xu 2014[29]										○○○(grey)	○○○(grey)					
	**Instructional**	Aikens 2010[16,B]											○○(grey) ○○					

	**Support**	Aikens 2012[5]		■■■ ■■ ↓									■■■ ■■ ↓					
		Burchinal 2014[7]											★ ■■● ●					
		Gosse 2014[31]						■										
		Guo 2010[30,A]											■■				■■	
		Hamre 2014[10]				■												
		Logan 2011[32] - Head Start						▰										
		Logan 2011[32] - State Funded					■											
		Peisner-Feinberg 2008[11] - NC 07/08											❖					
		Peisner-Feinberg 2013[12]			■													
		Weiland 2013[13]											■■■					
		West 2010[17,B]											○○					
		Xu 2014[29]										○	○					
	**Instructional Support (Language Modeling not included)**	Downer 2012[9] - DLL[A]	■		■													
		Downer 2012[9] - Latino[A]	■		■													
		Reid 2013[15,A]							○				○					
**CLASS Factors**	**Emotional Climate**	Burchinal 2010[18,A]							■■ ❖❖				■■ ❖❖					
		Burchinal 2012[19] - Whole Sample[A]											■					
		Burchinal 2012[19] - Spanish-English											★					

		Testing[A]																
		Burchinal 2012[19] - Spanish-Spanish Testing[A]										★ (grey)						
		Dotterer 2012[20,A]	■ (grey)		■ (grey)					■ (grey)			■ (grey)					
		Early 2006[21,A]			★				★				★					
		Howes 2008[22,A]	■★ (grey)		■★ (grey)				■★ (grey)				■★ (grey)					
		Mashburn, Pianta 2008[23,A]			■ (grey)				■ (grey)				■ (grey)					
		Mashburn 2009[33,A]							■				■					
	**Instructional Climate**	Burchinal 2008[24,A]	★ (grey)						★ (grey)				★ (grey)					
		Burchinal 2010[18,A]							■■ ❖❖				■■ ❖❖ (grey)					
		Burchinal 2011[3] - NCEDL[A]											⌘ (grey)					
		Burchinal 2012[19] - Whole Sample[A]											■ (grey)					
		Burchinal 2012[19] - Spanish English Testing[A]											★ (grey)					
		Burchinal 2012[19] - Spanish-Spanish Testing[A]											★ (grey)					
		Burchinal 2012[19] - Whole Sample[A]											■ (grey)					
		Dang 2011[25] - NCEDL[A]											■					
		Dotterer 2012[20,A]	■		■ (grey)					■			■					
		Early 2006[21,A]			★				★				★					
		Howes 2008[22,A]	■★		■★ (grey)				■★				■ ★ (grey ★)					
		Keys 2013[26] - NCEDL[A]											■					
		Mashburn, Pianta 2008[23,A]			■				■				■					

**Subscales**	**Behaviour Management**	Dobbs-Oates 2011[34,X]											■■■ (ns) ■				■	
		Guo 2014 [35,U]											■ (ns)					
	**Language Modeling**	Aikens 2010[16,B]											O O *O O*					
		Aikens 2012[5]		■■■ (ns) ■■ (ns) 🡻 (ns)									■■■ (ns) ■ (ns) ■ 🡻 (ns)					
		Hamre 2010[28]								■ (ns)			■ (ns)	■ (ns)	■ (ns)			
	**Literacy Focus**	Hamre 2010[28]								■			■ (ns)	■	■ (ns)			

Legend for Table

**Significant and Positive**	**Significant and Negative**	**Nonsignificant**	**Statistic**
★	*★*	★ (ns)	**r - Zero Order Pearson's Correlation**
O	*O*	O (ns)	**Beta**
■	*■*	■ (ns)	**B (Unstandardized Coefficient)**
❖	*❖*	❖ (ns)	**T-Test**
⌘	*⌘*	⌘ (ns)	**Partial Correlation**
●	*●*	● (ns)	**F-Ratio**
🡻	*🡻*	🡻 (ns)	**Effect Size**

Abbreviations: Total Score (factor ver.) = Total Score for Emotional Climate and Instructional Climate; Total Score (9 scale ver.) = Total Score with Language Modeling and Literacy Focus subscales not included. Total Score (8 scale ver.) = Total score with Regard for Student Perspectives, Language Modeling, and Literacy Focus subscales not included. For more details please see Table 2 in the manuscript.

[a]This paper is one of a series of Meta-Analyses and Systematic Reviews assessing the relationship between child care quality and children's outcomes; therefore, superscript letters below are in reference to various large databases that samples in these papers were drawn from. These letters have been kept consistent across the series for our readers.

[b]Samples within papers are described in more detail in the manuscript in Table 2.

[c]Acronyms for child outcomes are listed in Supplemental Information 2.

[d]Identifying Letters (also refers to Letter Knowledge, Letter-Naming Test, and Naming Letters).

[e]Hamre, 2014[10] used only Phonological Awareness and Print Knowledg subscales.

[A]National Center for Early Development and Learning Dataset (NCEDL, 2002, 2004); [B]Head Start Family and Children Experiences Survey (FACES, 2006) Cohort; [M]Head Start Family and Children Experiences Survey (FACES, 2009) Cohort; [X]Ohio Virginia (2005-2006, 2006-2007).

A Systematic Review and Meta-Analysis of a Measure of Staff/Child Interaction Quality (the Classroom Assessment Scoring System) in Early Childhood Education and Care Settings and Child Outcomes

Supplemental Information 4
Systematic Review Results: All Outcomes

Table C. CLASS Predicting all Language Outcomes T to W

CLASS Measurement Level		STUDY[a, b]	LANGUAGE[c]								
			TOPEL - Total Score[e]	TOPEL - Phonological Awareness	TOPEL - Print Knowledge	WJ - Letter Word ID	WJ - Picture Vocabulary	WJ - Rhyming	WJ - Sound Awareness	WJ - Spelling	WJ - Word Attack
**Total Score**	**Factor Version**	Burchinal 2011[3] - NCEDL[A]				▰■⌘					
	**9 Scale Version**	Sabol 2013[4,A]						★○			
**CLASS Domains**	**Classroom Organization**	Aikens 2012[5,M]				■■■■■ ↓				■■■■■	
		Bulotsky-Shearer 2014[6]				■★	■★				
		Burchinal 2014[7]		●●■■★			●●■■ ★				
		Hamre 2014[10]	■								
		Peisner-Feinberg 2008[11] - NC 07/08		❖	❖	❖					
		Peisner-Feinberg 2013[12]				■	■		■		■
		Xu 2014[29]	○○								
	**Emotional Support**	Bulotsky-Shearer 2014[6]				■★	■★				
		Burchinal 2014[7]		■■●●★		■■●●★					
		Curby 2013[14,A]						■■ ★			
		Hamre 2014[10]	■								

A Systematic Review and Meta-Analysis of a Measure of Staff/Child Interaction Quality (the Classroom Assessment Scoring System) in Early Childhood Education and Care Settings and Child Outcomes

		Peisner-Feinberg 2008[11] - NC 07/08		❖	❖	❖					
		Peisner-Feinberg 2013[12]				■	■		■		■
		Xu 2014[29]	○○○								
	**Instructional Support**	Aikens 2010[16,B]				○○					
		Aikens 2012[5]				■■■■■⬇				■■■■■	
		Bulotsky-Shearer 2014[6]	🕵			■★	■★				
		Burchinal 2014[7]		■■●●★		■■●●★					
		Hamre 2014[10]	■								
		Peisner-Feinberg 2008[11] - NC 07/08		❖	❖	❖					
		Peisner-Feinberg 2013[12]				■	■		■		■
		West 2010[17,B]				○○					
		Xu 2014[29]	○								
**CLASS Factors**	**Emotional Climate**	Burchinal 2010[18,A]				■■❖❖					
		Burchinal 2012[19] - Whole Sample[A]				■					
		Burchinal 2012[19] - Spanish-English Testing[A]				★					
		Burchinal 2012[19] - Spanish-Spanish Testing[A]				★					
		Dotterer 2012[20,A]						■			
		Early 2006[21,A]						★			
		Howes 2008[22,A]									
		Mashburn, Pianta 2008[23,A]						■			
	**Instructional Climate**	Burchinal 2010[18,A]				■■❖❖					
		Burchinal 2011[3] - NCEDL[A]				⌘					
		Burchinal 2012[19] - Whole Sample[A]				■					
		Burchinal 2012[19] - Spanish English Testing[A]				★					
		Burchinal 2012[19] - Spanish-Spanish Testing[A]				★					
		Dotterer 2012[20,A]						■			
		Early 2006[21,A]						★			
		Mashburn, Pianta 2008[23,A]						■			

**Subscale**	**Language Modeling**	Aikens 2010[16,B]				○○					
		Aikens 2012[5]				■■■■■🡻				■■■■■	

Legend for Table			
**Significant and Positive**	**Significant and Negative**	**Nonsignificant**	**Statistic**
★	★	★	**r - Zero Order Pearson's Correlation**
○	○	○	**Beta**
■	■	■	**B (Unstandardized Coefficient)**
❖	❖	❖	**T-Test**
⌘	⌘	⌘	**Partial Correlation**
🡻	🡻	🡻	**Effect Size**
●	●	●	**F-Ratio**

Abbreviations: Total Score (factor ver.) = Total Score for Emotional Climate and Instructional Climate; Total Score (9 scale ver.) = Total Score with Language Modeling and Literacy Focus subscales not included. Total Score (8 scale ver.) = Total score with Regard for Student Perspectives, Language Modeling, and Literacy Focus subscales not included. For more details please see Table 2 in the manuscript.

[a]This paper is one of a series of Meta-Analyses and Systematic Reviews assessing the relationship between child care quality and children's outcomes; therefore, superscript letters below are in reference to various large databases that samples in these papers were drawn from. These letters have been kept consistent across the series for our readers.

[b]Samples within papers are described in more detail in the manuscript in Table 2 in the manuscript..

[c]Acronyms for child outcomes are listed in Supplemental Information 2.

[A]National Center for Early Development and Learning Dataset (NCEDL, 2002, 2004); [B]Head Start Family and Children Experiences Survey (FACES, 2006) Cohort; [M]Head Start Family and Children Experiences Survey (FACES, 2009) Cohort; [X]Ohio Virginia (2005-2006, 2006-2007).

A Systematic Review and Meta-Analysis of a Measure of Staff/Child Interaction Quality (the Classroom Assessment Scoring System) in Early Childhood Education and Care Settings and Child Outcomes

Supplemental Information 4
Systematic Review Results: All Outcomes

Table D. CLASS Predicting all Social Emotional Outcomes, Positive Behaviour and Problem Behaviour

CLASS Measurement Level		STUDY[a, b]	Positive Behaviour [c]									Problem Behaviour						
			ASBI-Prosocial Behaviour	ECLS-K Approaches to Learning	Learning Self-Efficacy	Pencil Tapping Task	Social Skills (author created)	SSIS/SSRS – Social Skills	SSBPS – Social Competence	STRS - Closeness	TCRS – Social Competence	Behaviour Problems (author created)	SSIS – Internalizing Problems	SSIS – Externalizing Problems	SSIS/SSRS - Behaviours Problems	SSBPS – Behaviour Problems	STRS - Conflict	TCRS – Behaviour Problems
**Total Score**	**Factor Version**	Burchinal 2011[3] – NCEDL[A]									■■⌘							■■ ⌘
	**9 Scale Version**	Sabol 2013[4,A]									O★							O★
**CLASS Domains**	**Classroom Organization**	Aikens 2012[5,M]		■■ ■■ ■↓		■■ ■■ ■↓		■■ ■■ ■↓				■ ■ ■ ■ ■ ↓						
		Burchinal 2014[7]					★●● ■■					★ ●● ■▰						
		Downer 2012[9] – DLL[A]									■							▰
		Downer 2012[9] – Latino[A]									■							▰

		Hamre 2014[10]				■				g■							g■	
		Hestenes 2015[36]			g■			g■					g■	g■				
		Peisner-Feinberg 2008[11] - NC 07/08						❖							g❖			
		Peisner-Feinberg 2013[12]						g■							g■			
		Weiland 2013[13]				■ g■<br>g■												
	Emotional Support	Aikens 2012[5,M]		g■ g■<br>g■ g■<br>g■<br>g↓		g■ g■<br>g■ g■<br>g■ g↓<br>↓		g■ g■<br>g■ g■<br>g■<br>g↓				g■<br>g■<br>g■<br>g■<br>g■<br>g↓						
		Burchinal 2014[7]						g★ ● g●<br>g■ g■				★<br>● g●<br>g■ g■						
		Curby 2013[14,A]									★ ■ g■							▰ ★<br>g■
		Downer 2012[9] – DLL[A]									■							g■
		Downer 2012[9] – Latino[A]									■							g■
		Hamre 2014[10]				▰				g■							g■	
		Hestenes 2015[36]			g■			g■					■	g■				
		Johnson 2013[37]	g■															
		Peisner-Feinberg 2008[11] - NC 07/08						g❖							g❖			
		Peisner-Feinberg 2013[12]						g■							g■			
		Reid 2013[15,A]									g○							
		Weiland 2013[13]				■ ■<br>g■												
	Instructional Support	Aikens 2010[16,B]						g○ g○				g○<br>g○						
		Burchinal 2014[7]					g★<br>● g●					g★<br>g●						

(g = symbol printed in grey; unmarked symbols are black)

A Systematic Review and Meta-Analysis of a Measure of Staff/Child Interaction Quality (the Classroom Assessment Scoring System) in Early Childhood Education and Care Settings and Child Outcomes

							■■					● ▰ ■						
		Hamre 2014[10]				■				■							■	
		Hestenes 2015[36]			■			■					■	■				
		Peisner-Feinberg 2008[11] - NC 07/08						❖							❖			
		Peisner-Feinberg[12] 2013						■							■			
		Weiland 2013[13]				■■ ▰												
		West 2010[17,B]						○							○			
	Instructional Support (Language Modeling not included)	Downer 2012[9] – DLL[A]									■							■
		Downer 2012[9] – Latino[A]									■							■
		Reid 2013[15,A]									○							
CLASS Factors	Emotional Climate	Burchinal 2010[18,A]									■■❖ ❖							▰■ ❖❖
		Burchinal 2011[3] – NCEDL[A]									⌘							⌘
		Howes 2008[22,A]							★■							★■		
		Keys 2013[26] - NCEDL[A]									■							■
		Mashburn, Pianta 2008[23,A]									■							▰
	Instructional Climate	Burchinal 2008[24,A]									★							★
		Burchinal 2010[18,A]									■■❖ ❖							■▰ ❖❖
		Howes 2008[22,A]							★■								★■	
		Mashburn, Pianta 2008[23,A]									■							■

A Systematic Review and Meta-Analysis of a Measure of Staff/Child Interaction Quality (the Classroom Assessment Scoring System) in Early Childhood Education and Care Settings and Child Outcomes

Subscales	**Language Modeling**	Aikens 2010[16,B]						○○				○ ○						
	**Positive Climate**	Aikens 2012[5,M]		■■ ■■ ■ ↓		■■ ■■ ■ ↓		■■ ■■ ■ ↓				■ ■ ■ ■ ■ ↓						

Legend for Table

Significant & Positive	Significant & Negative	Nonsignificant	Statistic
★	★	★	**r - Zero Order Pearson's Correlation**
○	○	○	**Beta**
■	■	■	**B (Unstandardized Coefficient)**
❖	❖	❖	**T-Test**
⌘	⌘	⌘	**Partial Correlation**
●	●	●	**F-Ratio**
↓	↓	↓	**Effect Size**

Abbreviations: Total Score (factor ver.) = Total Score for Emotional Climate and Instructional Climate; Total Score (9 scale ver.) = Total Score with Language Modeling, Literacy Focus subscales not included. For more details please see Table 2 in the manuscript.

[a]This paper is one of a series of Meta-Analyses and Systematic Reviews assessing the relationship between child care quality and children's outcomes; therefore, superscript letters below are in reference to various large databases that samples in these papers were drawn from. These letters have been kept consistent across the series for our readers.

[b]Samples within papers are described in more detail in the manuscript in Table 2.

[c]Acronyms for child outcomes are listed in Supplemental Information 2.

[A]National Center for Early Development and Learning Dataset (NCEDL, 2002, 2004); [B]Head Start Family and Children Experiences Survey (FACES, 20

A Systematic Review and Meta-Analysis of a Measure of Staff/Child Interaction Quality (the Classroom Assessment Scoring System) in Early Childhood Education and Care Settings and Child Outcomes

References

1. Carlson SM. Developmentally sensitive measures of executive function in preschool children. *Dev Neuropsychol*. 2005;28(2):595-616. doi:10.1207/s15326942dn2802_3.

2. Gathercole S, Pickering S. Working memory deficits in children with low achievements in the national curriculum at 7 years of age. *Br J Educ Psychol*. 2000;70(2):177-194. doi:10.1348/000709900158047.

3. Burchinal M, Kainz K, Cai K. How well do our measures of quality predict child outcomes? A meta-analysis and coordinated analysis of data from large-scale studies of early childhood settings. In: Zaslow M, Martinez-Beck I, Tout K, Halle T, eds. *Quality Measurement in Early Childhood Settings*. Baltimore, MD: Paul H Brookes Publishing; 2011:11-31. http://www.researchconnections.org/childcare/resources/21062.

4. Sabol T, Soliday Hong S, Pianta R, Burchinal M. Can rating pre-k programs predict children's learning? *Science*. 2013;341(6148):845-846. doi:10.1126/science.1233517.

5. Aikens N, Moiduddin E, Xue Y, Tarullo L, West J. Data tables for child outcomes and classroom quality in FACES 2009 report. OPRE Report 2012- 37b. Washington, DC: Administration for Children and Families; 2012:1-200. http://www.acf.hhs.gov/sites/default/files/opre/data_tables_for_child_outcomes_and_classroom_quality_in_faces_2009.pdf. Accessed July 13, 2015.

6. Bulotsky-Shearer R, Bell E, Carter T, Dietrich S. Peer play interactions and learning for low-income preschool children: the moderating role of classroom quality. *Early Educ Dev*. 2014;25(6):815-840. doi:10.1080/10409289.2014.864214.

7. Burchinal M, Vernon-Feagans L, Vitiello V, Greenberg M, The Family Life Project Key Investigators. Thresholds in the association between child care quality and child outcomes in rural preschool children. *Early Child Res Q*. 2014;29(1):41-51. doi:10.1016/j.ecresq.2013.09.004.

8. Dominguez X, Vitiello V, Maier M, Greenfield D. A longitudinal examination of young children's learning behavior: child-level and classroom-level predictors of change throughout the preschool year. *Sch Psychol Rev*. 2010;39(1):29-47.

9. Downer J, López M, Grimm K, Hamagami A, Pianta R, Howes C. Observations of teacher–child interactions in classrooms serving Latinos and dual language learners: applicability of the classroom assessment scoring system in diverse settings. *Early Child Res Q*. 2012;27(1):21-32. doi:10.1016/j.ecresq.2011.07.005.

10. Hamre B, Hatfield B, Pianta R, Jamil F. Evidence for general and domain-specific elements of teacher-child interactions: associations with preschool children's development. *Child Dev*. 2014;85(3):1257-1274. doi:10.1111/cdev.12184.
11. Peisner-Feinberg E, Schaaf J. Evaluation of the North Carolina More at Four Pre-Kindergarten Program : performance and progress in the seventh Year (2007-2008). Chapel Hill, NC: FPG Child Development Institute; 2008. http://fpg.unc.edu/sites/fpg.unc.edu/files/resources/reports-and-policy-briefs/MAF_Yr7_full_report.pdf. Accessed July 13, 2015.
12. Peisner-Feinberg E, Schaaf J, LaForett D. Children's growth and classroom experiences in Georgia's pre-k program: findings from the 2011–2012 evaluation study. Chapel Hill, NC: FPG Child Development Institute; 2013. http://files.eric.ed.gov/fulltext/ED541933.pdf.
13. Weiland C, Ulvestad K, Sachs J, Yoshikawa H. Associations between classroom quality and children's vocabulary and executive function skills in an urban public prekindergarten program. *Early Child Res Q*. 2013;28(2):199-209. doi:10.1016/j.ecresq.2012.12.002.
14. Curby T, Brock L, Hamre B. Teachers' emotional support consistency predicts children's achievement gains and social skills. *Early Educ Dev*. 2013;24(3):292-309. doi:10.1080/10409289.2012.665760.
15. Reid J, Ready D. High-quality preschool: the relationship between the socioeconomic composition of preschool classrooms and children's learning. *Early Educ Dev*. 2013;24(8):1082-1111. doi:10.1080/10409289.2012.757519.
16. Aikens N, Tarullo L, Hulsey L, Ross C, West J, Xue Y. ACF-ORPRE Report: a year in Head Start: children, families and programs. Washington DC: Administration for Children and Families; 2010:1-36. http://files.eric.ed.gov/fulltext/ED517213.pdf. Accessed July 13, 2015.
17. West J, Malone L, Hulsey L, Aikens N, Tarullo L. ACF-OPRE Report: Head Start children go to kindergarten. Washington, DC: U.S. Department of Health and Human Services, Administration for Children and Families, Office of Planning, Research and Evaluation; 2010. http://www.acf.hhs.gov/sites/default/files/opre/hs_kindergarten.pdf.
18. Burchinal M, Vandergrift N, Pianta R, Mashburn A. Threshold analysis of association between child care quality and child outcomes for low-income children in pre-kindergarten programs. *Early Child Res Q*. 2010;25(2):166-176. doi:10.1016/j.ecresq.2009.10.004.
19. Burchinal M, Field S, López M, Howes C, Pianta R. Instruction in Spanish in pre-kindergarten classrooms and child outcomes for English language learners. *Early Child Res Q*. 2012;27(2):188-197. doi:10.1016/j.ecresq.2011.11.003.

20. Dotterer A, Burchinal M, Bryant D, Early D, Pianta R. Universal and targeted pre-kindergarten programmes: a comparison of classroom characteristics and child outcomes. *Early Child Dev Care*. 2012;183(7):931-950. doi:10.1080/03004430.2012.698388.

21. Early D, Bryant D, Pianta R, et al. Are teachers' education, major, and credentials related to classroom quality and children's academic gains in pre-kindergarten? *Early Child Res Q*. 2006;21(2):174-195. doi:10.1016/j.ecresq.2006.04.004.

22. Howes C, Burchinal M, Pianta R, et al. Ready to learn? children's pre-academic achievement in pre-kindergarten programs. *Early Child Res Q*. 2008;23(1):27-50. doi:10.1016/j.ecresq.2007.05.002.

23. Mashburn A, Pianta R, Hamre B, et al. Measures of classroom quality in prekindergarten and children's development of academic, language, and social skills. *Child Dev*. 2008;79(3):732-749. doi:10.1111/j.1467-8624.2008.01154.x.

24. Burchinal M, Howes C, Pianta R, et al. Predicting child outcomes at the end of kindergarten from the quality of pre-kindergarten teacher–child interactions and instruction. *Appl Dev Sci*. 2008;12(3):140-153. doi:10.1080/10888690802199418.

25. Dang T, Farkas G, Burchinal M, et al. Preschool center quality and school readiness: quality main effects and variation by demographic and child characteristics. Society for Research on Educational Effectiveness; 2011. http://eric.ed.gov/?id=ED519004. Accessed July 13, 2015.

26. Keys T, Farkas G, Burchinal M, et al. Preschool center quality and school readiness: quality effects and variation by demographic and child characteristics. *Child Dev*. 2013;84(4):1171-1190. doi:10.1111/cdev.12048.

27. McGinty A, Justice L, Piasta S, Kaderavek J, Fan X. Does context matter? explicit print instruction during reading varies in its influence by child and classroom factors. *Early Child Res Q*. 2012;27(1):77-89. doi:10.1016/j.ecresq.2011.05.002.

28. Hamre B, Justice L, Pianta R, et al. Implementation fidelity of MyTeachingPartner literacy and language activities: association with preschoolers' language and literacy growth. *Early Child Res Q*. 2010;25(3):329-347. doi:10.1016/j.ecresq.2009.07.002.

29. Xu Y, Chin C, Reed E, Hutchinson C. The effects of a comprehensive early literacy project on preschoolers' language and literacy skills. *Early Child Educ J*. 2014;42(5):295-304. doi:10.1007/s10643-013-0613-6.

30. Guo Y, Piasta S, Justice L, Kaderavek J. Relations among preschool teachers' self-efficacy, classroom quality, and children's language and literacy gains. *Teach Teach Educ*. 2010;26(4):1094-1103. doi:10.1016/j.tate.2009.11.005.

31. Gosse C, McGinty A, Mashburn A, Hoffman L, Pianta R. The role of relational and instructional classroom supports in the language development of at-risk preschoolers. *Early Educ Dev*. 2014;25(1):110-133. doi:10.1080/10409289.2013.778567.

32. Logan J, Piasta S, Justice L, Schatschneider C, Petrill S. Children's attendance rates and quality of teacher-child interactions in at-risk preschool classrooms: contribution to children's expressive language growth. *Child Youth Care Forum*. 2011;40(6):457-477. doi:10.1007/s10566-011-9142-x.

33. Mashburn A, Justice L, Downer J, Pianta R. Peer effects on children's language achievement during pre-kindergarten. *Child Dev*. 2009;80(3):686-702. doi:10.1111/j.1467-8624.2009.01291.x.

34. Dobbs-Oates J, Kaderavek J, Guo Y, Justice L. Effective behavior management in preschool classrooms and children's task orientation: enhancing emergent literacy and language development. *Early Child Res Q*. 2011;26(4):420-429. doi:10.1016/j.ecresq.2011.02.003.

35. Guo Y, Tompkins V, Justice L, Petscher Y. Classroom age composition and vocabulary development among at-risk preschoolers. *Early Educ Dev*. 2014;25(7):1016-1034. doi:10.1080/10409289.2014.893759.

36. Hestenes L, Kintner-Duffy V, Wang Y, et al. Comparisons among quality measures in child care settings: understanding the use of multiple measures in North Carolina's QRIS and their links to social-emotional development in preschool children. *Early Child Res Q*. 2015;30(Part B):199-214. doi:10.1016/j.ecresq.2014.06.003.

37. Johnson S, Seidenfeld A, Izard C, Kobak R. Can classroom emotional support enhance prosocial development among children with depressed caregivers? *Early Child Res Q*. 2013;28(2):282-290. doi:10.1016/j.ecresq.2012.07.003.
