# Supplementary material for: A Systematic Review and Meta-Analysis of a Measure of Staff/Child Interaction Quality (the Classroom Assessment Scoring System) in Early Childhood Education and Care Settings and Child Outcomes
Source: PLoS One. 2016 Dec 30;11(12):e0167660. doi: 10.1371/journal.pone.0167660 (PMC5201239; doi:10.1371/journal.pone.0167660)
Supplement: S5 File — (PDF) [file pone.0167660.s005.pdf]

A Systematic Review and Meta-Analysis of a Measure of Staff/Child Interaction Quality (the Classroom Assessment Scoring System) in Early Childhood Education and Care Settings and Child Outcomes

Supplemental Information 5

**List of Acronyms Used in the Description of Studies**	
**Acronym Category**	**Full Name**
**Journals**	Applied Developmental Science (ADS)<br>Child Development (CD)<br>Child & Youth Care Forum (CYCF)<br>Early Child Development and Care (ECDC)<br>Early Education and Development (EED)<br>Early Childhood Research Quarterly (ECRQ)<br>School Psychology Review (SPR)<br>Teaching and Teacher Education (T&TE)
**Large Samples**	Head Start Family and Children Experiences Study (FACES)<br>National Center for Early Development (NCEDL)<br>North Carolina (NC)<br>Study of State-Wide Early Education Programs (SWEEP)
**Covariates**	Applied Problems (AP)<br>Bachelor of Arts (BA)<br>Developmentally Appropriate Beliefs (DAP)<br>Dual Language Learner (DLL)<br>Early Childhood Education (ECE)<br>Environment Rating Scales (ECERS)<br>Environment Rating Scales-Revised (ECERS-R)<br>English Language Learner (ELL)<br>Head Start (HS)<br>Home Observation of the Measurement of the Environment (HOME)<br>Masters of Arts (MA)<br>Peabody Picture Vocabulary Test (PPVT)<br>Relational Support (RS)<br>Socio-Economic Status (SES)<br>Standard Deviation (SD)<br>Woodcock Johnson Achievement Test (WJ)
